# Defining the content and delivery of an intervention to Change AdhereNce to treatment in BonchiEctasis (CAN-BE): a qualitative approach incorporating the Theoretical Domains Framework, behavioural change techniques and stakeholder expert panels

**DOI:** 10.1186/s12913-015-1004-z

**Published:** 2015-08-22

**Authors:** Amanda R. McCullough, Cristín Ryan, Brenda O’Neill, Judy M. Bradley, J. Stuart Elborn, Carmel M. Hughes

**Affiliations:** Clinical and Practice Research Group, School of Pharmacy, Queen’s University Belfast, Belfast, UK; Centre for Research in Evidence-Based Practice, Bond University, Gold Coast, Australia; Northern Ireland Clinical Research Facility, Queen’s University Belfast and The Wellcome Trust-Wolfson Northern Ireland Clinical Research Facility, Belfast City Hospital, Belfast, UK; Centre for Infection and Immunity, School of Medicine, Dentistry and Biomedical Sciences, Queen’s University Belfast, Belfast, UK

## Abstract

**Background:**

Low patient adherence to treatment is associated with poorer health outcomes in bronchiectasis. We sought to use the Theoretical Domains Framework (TDF) (a framework derived from 33 psychological theories) and behavioural change techniques (BCTs) to define the content of an intervention to change patients’ adherence in bronchiectasis (Stage 1 and 2) and stakeholder expert panels to define its delivery (Stage 3).

**Methods:**

We conducted semi-structured interviews with patients with bronchiectasis about barriers and motivators to adherence to treatment and focus groups or interviews with bronchiectasis healthcare professionals (HCPs) about their ability to change patients’ adherence to treatment. We coded these data to the 12 domain TDF to identify relevant domains for patients and HCPs (Stage 1). Three researchers independently mapped relevant domains for patients and HCPs to a list of 35 BCTs to identify two lists (patient and HCP) of potential BCTs for inclusion (Stage 2). We presented these lists to three expert panels (two with patients and one with HCPs/academics from across the UK). We asked panels who the intervention should target, who should deliver it, at what intensity, in what format and setting, and using which outcome measures (Stage 3).

**Results:**

Eight TDF domains were perceived to influence patients’ and HCPs’ behaviours: Knowledge, Skills, Beliefs about capability, Beliefs about consequences, Motivation, Social influences, Behavioural regulation and Nature of behaviours (Stage 1). Twelve BCTs common to patients and HCPs were included in the intervention: Monitoring, Self-monitoring, Feedback, Action planning, Problem solving, Persuasive communication, Goal/target specified:behaviour/outcome, Information regarding behaviour/outcome, Role play, Social support and Cognitive restructuring (Stage 2). Participants thought that an individualised combination of these BCTs should be delivered to all patients, by a member of staff, over several one-to-one and/or group visits in secondary care. Efficacy should be measured using pulmonary exacerbations, hospital admissions and quality of life (Stage 3).

**Conclusions:**

Twelve BCTs form the intervention content. An individualised selection from these 12 BCTs will be delivered to all patients over several face-to-face visits in secondary care. Future research should focus on developing physical materials to aid delivery of the intervention prior to feasibility and pilot testing. If effective, this intervention may improve adherence and health outcomes for those with bronchiectasis in the future.

**Electronic supplementary material:**

The online version of this article (doi:10.1186/s12913-015-1004-z) contains supplementary material, which is available to authorized users.

## Background

Bronchiectasis is a chronic lung disease with a rising prevalence in the United States, of 8.7 % per year between 2000 and 2007 [1]. Patients with this condition experience debilitating symptoms and impaired quality of life [2]. Treatments are burdensome and time-consuming, with patients prescribed an average of 12 medications as well as airway clearance techniques to clear lung secretions [3]. Less than 20 % of patients are adherent to all aspects of treatment [3]. Patients who are non-adherent to inhaled antibiotics have four pulmonary exacerbations per year, compared to 2.6 per year in adherent patients [3], indicating a need to optimise adherence to treatment in this population. There are no evidence-based interventions currently available to change adherence for patients with bronchiectasis [4].

A growing body of evidence advocates the use of psychological theories in the development of interventions to change behaviour [5–7]. A lack of psychological theory use in the development of adherence interventions may limit the efficacy and implementation of these interventions in chronic respiratory disease [8–10]. Consequently, recent guidelines emphasise the need to report three aspects of behaviour change interventions [11]: (1) use of psychological theory to identify the factors which influence the target behaviour change (i.e., *mechanism of action*); (2) the active ingredients of behaviour change interventions (i.e., the content or *what* was delivered); and, (3) *how* this was delivered (i.e., who the intervention targeted, who delivered it, at what intensity, in what format and setting [12]).

The Theoretical Domains Framework (TDF) [13] can be used to define the mechanism of action and to choose behaviour change techniques (BCTs) (“active ingredients”) to include in an intervention [7, 14–17]. Two versions of the TDF exist [13, 18] but only the 12 domain version has been used at the time of this study to select BCTs to include in a behaviour change intervention [13, 16]. Three compilations of BCTs have been published [7, 14, 17] but only one has been used with the TDF to define the content of a behaviour change intervention [7, 16]. Defining the content in this way does not indicate how this content should be delivered. Involving key stakeholders represents a potentially useful method of doing this for an intervention to change adherence in bronchiectasis [16, 19]. Therefore, the aim of this study was to use a theoretically driven approach, informed by stakeholders’ perspectives to develop an intervention that would focus on changing patient adherence to treatments in bronchiectasis.

## Methods

**Fig. 1:**
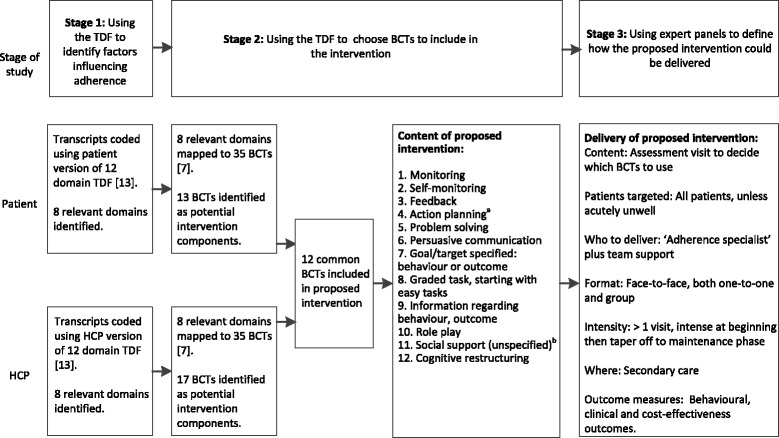
Summary of stages of data analysis, content and delivery of proposed intervention. ^a^Action planning replaced ‘Time management’ in the BCT Taxonomy [17]. ^b^Social support (unspecified) replaced ‘Motivational interviewing’ and ‘Social processes of encouragement, pressure, support’ in the BCT Taxonomy [17]. TDF: Theoretical domains framework. BCTs: Behavioural change techniques. HCP: Healthcare professional

This study consisted of three stages: to use the TDF to identify what factors influence patients’ adherence behaviour and identify what factors influenced healthcare professionals’ (HCPs) ability to change the adherence behaviour of patients. i.e., *mechanism of action of our intervention* (Stage 1); use data from Stage 1 to choose the BCTs i.e., the *active ingredients* to include in a proposed intervention (Stage 2); and, use expert panels of key stakeholders (patients, HCPs and academics) to define *how* the proposed intervention could be delivered including format and delivery, training of HCPs and commissioning of the proposed intervention in the future (Stage 3). Figure 1 illustrates the three stages of the study.

### Stage 1: using the TDF to identify what factors influenced adherence

#### Design

We completed two qualitative studies. Firstly, we interviewed patients with bronchiectasis (November 2011-April 2012). Design, sampling and materials used in this study have been reported in detail previously [20]. We re-analysed data collected from patient interviews using the TDF and will only discuss this re-analysis in this paper. Secondly, between January and June 2013, we completed semi-structured, mixed-discipline focus groups and one-to-one interviews with HCPs. One-to-one interviews were completed with those HCPs who were unable to attend focus groups.

#### Sample

We recruited patients with bronchiectasis with a history of *Pseudomonas aeruginosa* infection using maximum variation sampling as they approached the end of a related study [3, 20]. We recruited HCPs who cared for patients with bronchiectasis (nurses, physiotherapists, respiratory physicians, general practitioners [GPs], hospital and community pharmacists and psychologists) from the five health administrative bodies in Northern Ireland [Health and Social Care Trusts (HSC)]. Participants were recruited using a snowballing recruitment strategy [21]. We approached existing clinical contacts to participate in the study and/or to nominate others who might be interested. Recruitment was supported by the Northern Ireland Clinical Research Network (specifically the Respiratory Health and Primary Care Interest Groups) who approached potential participants (GPs and those in secondary care in Belfast and Western HSC Trusts). Participants were invited via email using an invitation letter and information sheet. Recruitment continued until data saturation was reached [21].

#### Materials and procedure

Both studies used semi-structured topic guides. The patient guide focused on the factors (barriers and motivators) influencing adherence to treatments and strategies to overcome these [20]. The HCP guide focused on knowledge of adherence to treatment, perceptions of factors influencing adherence, ability to change adherence and views on the important components of an intervention to change adherence in bronchiectasis (Additional file 1). Participants in both studies provided informed consent immediately prior to taking part. The researcher (AMcC) conducted one-to-one interviews with patients in their chosen location (hospital or own home) [20]. Two facilitators (AMcC and CR) conducted mixed discipline HCP focus groups in training rooms at seven hospital sites. Both facilitators were HCPs (physiotherapist and pharmacist) with experience and training in conducting qualitative research. The researcher (AMcC) interviewed those who were unable to participate in focus groups. These took place in the private offices of the interviewees.

#### Analysis

All interviews and focus groups were transcribed verbatim, anonymised and checked for accuracy by AMcC. All data were analysed using the 12 domain TDF [13] (Additional file 1: Tables S1 and S2), patient data were analysed using an adapted version (Additional file 1: Table S1). All transcripts were independently analysed by the researcher (AMcC) and a second member of the team (JB, BO’N, CH or CR). We coded sections of transcripts to the TDF domains. Each pair of researchers coded several transcripts then met to agree the approach to coding. The researcher (AMcC) ensured that all second coders were using the same approach; final coding for each domain was agreed between coders. The researcher (AMcC) analysed the content of each TDF domain for both patient and HCP domains to identify key themes emerging in each. We used this information to reach consensus on the relevance of each TDF domain to adherence behaviour for patients and HCPs. We agreed a domain to be ‘relevant’ if it was frequently coded and the content of the domain linked directly to the behaviour of interest [22].

### Stage 2: using the TDF to choose BCTs to include in the intervention

To identify BCTs for inclusion, three members of the team mapped the relevant TDF domains to a list of 35 BCTs using the following method [7] (a list of the 35 BCTs and their definitions are included in Additional file 2: Table S3). We independently scored whether we would use each BCT to target each of the 12 TDF domains using an adapted scoring system from Michie *et al.* [7] outlined in Table 1. Additional file 2: Tables S4 and S5 show the final mapping of TDF domains to BCTs for patients and HCPs.

**Table 1:** Scoring system for choosing potential BCTs to include in the intervention

Category for each BCT	Scoring for each BCT^a^
Agreed use	Two or more raters scored with a 2 or 3, except if the third rater scored a 0
Agreed non-use	Two or more raters scored with a 0
Disagreement	One rater scored with a 0 and two raters scored with a 2 or 3
Uncertain	All other cells in the matrix

^a^Three raters independently scored each BCT as 0-3, where 0 = no, 1 = possibly, 2 = probably and 3 = definitely

BCTs for which there was ‘agreed non-use’ were excluded. We ranked the remaining BCTs based on the number of TDF domains for which there was ‘agreed use’ or ‘agreed use’ plus ‘disagreement.’ (Additional file 2: Tables S4 and S5). We discussed, as a team, whether these BCTs should be included, using the following information to aid our discussion: (1) BCT ranking; (2) data on effectiveness of adherence interventions collected as part of a relevant systematic review [4]; (3) knowledge of the transcript content; (4) whether the BCT was included, excluded or changed in the BCT taxonomy which was published during the time this study was being conducted [17]. From this, we produced two refined lists of potential BCTs: one to change patient adherence behaviour and one to change HCPs’ ability to change adherence. We identified the common BCTs across the two lists and cross-referenced this final list of potential BCTs with the BCT taxonomy to check that the terminology and definitions used were up-to-date [17]. We adopted this approach of combining the two lists and cross-referencing with the BCT taxonomy following personal communications with health psychologists with specific expertise in applying the TDF and in BCT coding.

### Stage 3: using expert panels to define how the proposed intervention could be delivered

#### Design

In April and May 2014, we conducted three expert panels (patients with *P. aeruginosa* infection, patients without *P. aeruginosa* infection and one HCP/academic panel). Patients with a history of *P. aeruginosa* infection participated in a separate panel from those without *P. aeruginosa* to minimise potential cross-infection.

#### Sample

Patients were eligible if they had high resolution computed tomography diagnosed bronchiectasis and were prescribed treatments for bronchiectasis. Patients were recruited by their HCPs from across the five HSC Trusts, to gain a maximum variation sample based on gender, location and *P.aeruginosa* infection status. HCP and academics from across the United Kingdom were purposively sampled to obtain a range of expertise (bronchiectasis management, intervention development, commissioning of interventions and professional background). Potential participants were contacted via email and invited to participate.

#### Materials and procedure

We summarised the findings from Stages 1 and 2 plus two related studies (a systematic review of adherence interventions in chronic respiratory disease and a study measuring predictors of adherence in bronchiectasis [3, 4, 23]) and circulated these to participants two weeks prior to the expert panels taking place (Patient version in Additional file 3 and HCP version in Additional file 4). In advance of all panels, the research team used data from a relevant systematic review to decide if the BCTs delivered should be individualised to patients or treatments [4]. We prepared the following documents to use during the expert panels (Patient version in Additional file 5 and HCP version in Additional file 6): (1) a presentation outlining how the intervention was developed and an agenda for the panel; (2) a facilitators’ guide for use during the panels; (3) detailed summaries of relevant TDF domains from Stage 1; (4) a bronchiectasis-specific example for each of the 12 BCTs in the proposed intervention was generated by using the TDF findings from Stage 1 [7], BCT examples provided in the BCT taxonomy [17] and findings of a related systematic review [4]; and, (5) a list of questions to be discussed during the expert panel meetings based on those suggested by Davidson *et al.* [12] (Table 2). During all the panels, the researcher (AMcC) delivered the presentation described above. Following this, each panel completed two tasks (Table 2). Four researchers (AMcC, CR, BO’N and CH) facilitated the two patient panels. These took place on the same day (patients without *P.aeruginosa* infection in the morning and patients with history of *P.aeruginsa* in the afternoon). The single HCP panel was led by six facilitators (AMcC, CR, JB, BO’N, SE and CH) and took place over one day. Participants were split into three small groups each with specific questions to discuss (Table 2). In all panels, key discussion points were recorded by designated members of the research team.

**Table 2:** Questions explored with expert panels

Task 1: What do you think about our approach to intervention development?
Task 2 (small group task): Defining how the proposed intervention could be delivered *Two patient panels and Group 1 of HCP panel*
1. Which patients should the intervention be delivered to?
2. Who should deliver the intervention?
3. How often should the intervention be delivered?
4. For how long should the intervention be delivered?
5. What format should the intervention take?
6. Where should the intervention be delivered?
7. How would you know if the intervention was working? (patients only)
HCP/academic panel (Group 2)
1. Which healthcare professionals should the training be delivered to?
2. Who should deliver the healthcare professional training?
3. How often should the training take place?
4. How long should the training be?
5. What format should the healthcare professional training take?
6. Where should healthcare professional training be delivered?
HCP/academic panel (Group 3)
1. How do you commission services at the moment?
2. How do you commission training for staff at the moment?
3. Would improved adherence be enough to convince you that this intervention was worth implementing?
4. What would you need to convince you that this intervention was worth implementing?

#### Analysis

The research team conducted a de-brief session following all three expert panels. During this session, key findings from each panel were agreed. A copy of these findings was sent via post or email to the participants of the respective panels (Additional files 7 and 8). The researcher (AMcC) contacted participants via telephone or email to obtain feedback on this document which was added to the overall findings. The research team used these documents to agree a plan for how the intervention could be delivered in terms of content, who the intervention should target, who should deliver it, at what intensity, in what format and setting as well as the preferred outcome measures, approach to training and commissioning of the intervention in the future.

### Ethical considerations

Ethical approval for all studies was received from the Office for Research Ethics Northern Ireland (11/NI/0109 and 12/NI/0078). All HCP/academic participants who participated in interviews, focus groups or an expert panel received a Continuing Professional Development certificate. Patient and HCP/academic expert panel participants received a £100 honorarium for participating in an expert panel.

## Results

### Results from stage 1: using the TDF to identify what factors influenced adherence

Patient participants have been described previously [20]. Briefly, 16 patients with bronchiectasis and moderate lung impairment participated. Eight were non-adherent to treatment (inhaled antibiotics, other respiratory medicines and airway clearance) and eight were adherent to these treatments, based on scoring ≥ 80 % on a self-reported adherence questionnaire [20].

Seven focus groups of HCPs (*n* = 39, four to eight participants per group) and seven interviews with HCPs were completed (*n* = 7). Thirty-eight (83 %) participants were female with a mean [SD] of 19 [7] years since qualification. Nine participants worked in primary care (4 practice nurses, 3 GPs and 2 community pharmacists) and 37 in secondary care (16 nurses, 10 physiotherapists, 7 respiratory physicians, 2 pharmacists, 2 psychologists).

We identified the same eight domains as being relevant for patients and HCPs: Knowledge, Skills, Beliefs about capabilities, Beliefs about consequences, Motivation, Social Influences, Behavioural regulation and Nature of behaviours. Relevant TDF domains, their sub-themes, summary of domain content and sample quotes are included in Tables 3 and 4.

**Table 3:** Relevant TDF domains, sub-themes, summary of domain content and example quotes for interviews with patients with bronchiectasis (Stage 1)

Domain label	Sub-themes	Summary of domain content	Example quote
Knowledge	Knowledge of treatment	Patients had a broad understanding of most treatments but inhaled antibiotics were less well understood. Disease knowledge was vague and misinformed, particularly for knowledge of disease progression. In most cases, patients thought that having disease and treatment knowledge improved adherence.	*“I would have to find out exactly why I was put on it* (new treatment)*…I don’t think I would start taking it until I was satisfied.” (F10A)*
	Knowledge of disease		
Skills	Treatment skills	Most patients felt they had competent treatment skills. However, other patients did not feel they could competently complete airway clearance and this was a barrier to adherence. Patients frequently used self-monitoring skills to monitor symptoms and inform decisions about adherence either by reinforcing their current adherence behaviour or prompting a change in behaviour.	*“They* (physiotherapists) *taught me a method of just sitting up in bed and using the wedge and doing the drainage that way.” (F2NA)*
	Self-monitoring skills		
Beliefs about capabilities	Psychological capability	Patients were generally confident in using inhalers and oral medication. Nebulised medications and airway clearance were viewed to be more complex and some patients felt that they lacked the psychological capability to do these treatments, often reporting that doing treatments was monotonous. Patients thought their physical capability to adhere would change if they were older, had physical disabilities or were experiencing a pulmonary exacerbation.	*“I do do it myself but I don’t feel it’s as good as, em, someone doing it for you…you’re getting more attention than you’re giving it yourself.” (F6NA)*
	Physical capability		
Beliefs about consequences	Beliefs about necessity for treatment	Most patients believed that improved symptoms and quality of life were positive consequences of adherence. Those who reported a lack of perceived symptoms or symptomatic improvement following treatment had a lower perceived need for treatment. Some patients also believed that there were potential negative consequences of adherence, such as harm caused by taking medicines.	*“I don’t really need an antibiotic…if I stopped it for 2 weeks, 3 weeks, 4 weeks I wouldn’t feel any different.” (M3NA)*
	Beliefs about harm caused by treatment		
Motivation and goals	Intrinsic motivation	The majority of patients had high intrinsic motivation to adhere and prioritised adherence over other commitments. Some patients struggled with intrinsic motivation for airway clearance and inhaled antibiotics. Patients reported a desire to avoid negative consequences of non-adherence (hospital admission, pulmonary exacerbations and decline in quality of life) as goals that increased motivation to adhere.	*“I think, you’ve got to feel it within yourself that this is what you need to do* (adhere to treatment).” *(M14A)*
	Goal to avoid negative consequences		
Social influences	Trust in HCPs	Patients expressed an inherent trust in HCPs. They stated that the support of HCPs and other people with bronchiectasis built their confidence in managing their condition. Generally patients reported that their families were supportive but some did not want to be a burden on their families and did not involve them in their treatment. Family, social and working commitments were seen by some as barriers to adherence.	*“I do take them because they* (HCPs) *tell me to do that, you know, to take the whole course* (of oral antibiotics)*.” (M16A)*
	Social support		
	Competing social demands		
Behavioural regulation	Education	Patients suggested training on treatment skills, information on disease progression, reasons for doing treatment, expected treatment effects and negative consequences of non-adherence would encourage patients to adhere. Action planning and reminder strategies were suggested, with the caveat that the latter were only for those with difficulty remembering to do treatment. Access to and regular review by a specialist multidisciplinary team was thought to facilitate adherence. Several non-adherent patients thought that feedback on disease progression would facilitate adherence.	*“If somebody came along and said to me, ‘if you don’t take that Acapella® or use that Acapella® every morning and night, eh, you’re going to get worse, your bronchiectasis is going to get worse’ then would probably frighten me into taking it.” (M5NA)*
	Action planning		
	Reminder strategies		
	Regular review		
	Feedback on outcome		
Nature of behaviour	Routine	Most patients reported that adherence was something they did automatically. Most patients linked doing treatments to other activities such as mealtimes and bedtimes. Treatments that fell outside of the normal treatment routine or were more burdensome to integrate (e.g., airway clearance or inhaled antibiotics) were more likely to be missed.	*“I tend to do mine* (treatments) *with my early morning cup of tea and when I’m in bed at night.” (F12A)*

*F* Female, *M* Male, *1-16* Interview number, *A* adherent, *NA* non-adherent, *HCPs* healthcare professionals

**Table 4:** Relevant TDF domains, sub-themes, summary of domain content and example quotes for interviews and focus groups with HCPs (Stage 1)

Domain label	Sub-themes	Summary of domain content	Example quote
Knowledge	Clinical knowledge	Primary care HCPs lacked knowledge about bronchiectasis and its management. Better HCP disease knowledge was thought to translate to better patient disease knowledge. HCPs had a broad understanding of the potential barriers for patient adherence to treatment. Some HCPs stated that they did not know what to do to change patients’ adherence.	*“There’s probably not as much knowledge* (about bronchiectasis) *as there maybe should be.” (I4_PN)*
	Knowledge of adherence		
Skills	Interpersonal skills	HCPs stated that they used interpersonal skills such as questioning skills, building rapport, negotiation, problem-solving and persuasive communication to change adherence. Some HCPs felt that they lacked these skills. Some HCPs had formal postgraduate training in interpersonal skills, which they thought improved their ability to change patient adherence.	*“I think we would just rely on experience. I don’t think you’re ever given any specific training about adherence in any aspect.” (FG1_HP)*
Beliefs about capabilities	Confidence in ability to change adherence	HCPs had a general belief that they had limited control over changing patients’ adherence. Some lacked confidence in their ability to change adherence. Others felt confident in their ability to do this and those who did, tended to have completed extended communication skills training. Several participants appeared pessimistic about their ability to change their own behaviours around managing adherence, this was mainly linked to limitations due to environmental constraints.	*“I kind of dread the patient who I think isn’t compliant…the ability to honestly challenge a patient about their compliance without perhaps losing the relationship, the trust and stuff… It’s actually a quite challenging thing.” (FG5_D)*
	Confidence in ability to change own behaviour		
Beliefs about consequences	Positive consequences of changing adherence	HCPs believed that changing adherence could lead to positive consequences for the healthcare system and patients, through reduced hospital admissions and financial burden. They evaluated the need to change patients’ adherence based on their disease status. They only asked questions about adherence when patients were unwell. Some HCPs were concerned about the negative consequences of discussing adherence, such as sabotaging their relationship with that patient and a potentially increased workload.	*“*(Nurse’s name) *and I are trying to do the bronchiectasis service on top of our* (usual workload)*, which is a problem, so you know, in terms of chasing up adherence and chasing up patients to see what they’re doing isn’t always as possible.” (FG3_PT2)*
	Negative consequences of changing adherence		
Motivation and goals	Adherence not a priority	Changing adherence was not a priority for HCPs unless patients were unwell or there was a reason to suspect non-adherence. Bronchiectasis was not a priority for primary care participants, who viewed it as a secondary care problem.	*“If they’re not under the umbrella of asthma or COPD, well it doesn’t matter whether they’re seen or not* [laughs] *in theory.” (I1_PN)*
	Bronchiectasis not a priority for primary care		
Social influences	Influence of patients	Patients strongly influenced HCPs’ clinical decisions about adherence. Involving patients in decisions about treatment and adherence was viewed as being essential to changing adherence. Effective team working was thought to increase HCPs’ ability to manage adherence. A lack of team-working was evident between primary and secondary care.	*“If the GP changes something or if they* (patients) *go to hospital and something has changed…nobody lets the community pharmacist know…you sort of fall out of the loop a wee bit” (FG7_CP)*
	Influence of other HCPs		
Behavioural regulation	Patient-focused strategies	HCPs suggested patient-focused adherence strategies such as disease education, goal setting, action planning, problem-solving, social support, feedback about disease progression/adherence. System-focused strategies included a clear, multidisciplinary pathway across primary and secondary care. Suggested strategies to monitor adherence included electronically chipped inhalers, patient diaries, counting tablets and questioning patients about adherence. HCP-focused training on consultation skills was also recognised as being needed.	*“*(We need) *something that has everything in one book, you know, to explain medications, airway clearance, exercise, self-management, anxiety, depression all of those things in one booklet.” (FG7_PT2)*
	System-focused strategies		
	HCP-focused strategies		
Nature of behaviours	Changing adherence not part of routine care	HCPs stated that changing adherence was not part of current routine assessment and treatment for patients with bronchiectasis. However, they recognised that data on number of prescriptions are routinely collected by GP and pharmacy databases and thus, could be made available from primary care to secondary care to enable monitoring of dispensed items.	*“When you’re seeing a bronchiectasis patient you’re not automatically thinking of adherence.” (FG3_N1)*

*I* interview, *FG* focus group, *1-7* interview/focus group number, *PN* practice nurse, *HP* hospital pharmacist, *D* hospital doctor, *PT* physiotherapist, *CP* community pharmacist, *N* nurse, *HCP* healthcare professional

### Results from stage 2: using the TDF to choose BCTs to include in the intervention

Relevant TDF domains mapped against the 35 BCTs and ranked by number of domains targeted are shown in Additional file 2: Tables S4 and S5. For patients, 13 potential BCTs were identified as potential components of an intervention (Table 5). Eleven BCTs were excluded due to ‘agreed non-use’ and a further 11 were excluded following discussion within the team (reasons for exclusion are in Additional file 2: Table S6). For HCPs, 17 BCTs were identified as potential components of the intervention (Table 5). Eight BCTs were excluded due to ‘agreed non-use’ and a further 10 were excluded following discussion within the team (reasons for exclusion are in Additional file 2: Table S7). There were 12 common BCTs across patient and HCPs (Fig. 1). Following cross-checking of this final BCT list with the BCT taxonomy, we updated three of the included BCTs to terms used in the BCT taxonomy. Time management was changed to Action planning [17]. Motivational interviewing and Social processes of encouragement, pressure, support were both changed to Social support (unspecified) from the BCT taxonomy [17].

**Table 5:** Patient and HCP BCTs identified as potential intervention components (Stage 2)

Patient BCTs	HCP BCTs
Monitoring	Monitoring
Self-monitoring	Self-monitoring
Feedback	Feedback
Time management	Time management
Problem solving	Problem solving
Persuasive communication	Persuasive communication
Goal/target specified: behaviour or outcome	Goal/target specified: behaviour or outcome
Graded task, starting with easy tasks	Graded task, starting with easy tasks
Information regarding behaviour, outcome	Information regarding behaviour, outcome
Role play	Role play
Motivational interviewing	Social processes of encouragement, pressure and support
Cognitive restructuring	Cognitive restructuring
Shaping of behaviour	Rewards; incentives including self-evaluation
	Contract
	Increasing skills; problem solving, decision making, goal setting
	Self-talk
	Relapse prevention

*HCP* healthcare professional, *BCT* behavioural change techniques

### Results from stage 3: using expert panels to define how the proposed intervention could be delivered

Eleven patients (64 % female) participated across two panels (*P. aeruginosa* panel *n* = 5 and non-*P.aeruginosa* panel *n* = 6). Nine (78 % female) HCP/academics participated in a single panel (two nurses, three doctors, two physiotherapists, two psychologists). Four had expertise in bronchiectasis, three had experience in behaviour change interventions and two in both bronchiectasis and commissioning interventions. Prior to the panels, the research team decided that the proposed intervention should be able to be individualised to patients and treatments i.e., not all BCTs should be delivered to all patients but rather BCTs should reflect specific patient or treatment needs. All panels agreed with this approach and thought it would be of use to them. Panels’ views on the format and delivery of the intervention are summarised in Fig. 1. HCP/academics’ views on how training should be delivered are included in Table 6. HCP/academics thought that during evaluation of our proposed intervention pulmonary exacerbations, hospital admissions, lost work days and quality of life should be measured as these drive the commissioning of future services. Detailed findings from each of the three panels are included in Additional files 7 and 8.

**Table 6:** HCP/academic panel views on how HCPs should be trained to deliver the intervention (Stage 3)

Questions posed	HCPs/academic panel views
Who to train?	Lead HCP at each site. Whole MDT receive broader, less in-depth training
Who to deliver training?	Psychologist or another trained professional from outside the MDT
Intensity of training?	Lead at each site receiving 4 × 2 h sessions, 2 to 3 weeks apart. Mentoring and support via email or telephone ‘hotline.’ MDT should receive a half-day training session.
Format of training?	Problem-based learning in a group setting using role plays and case studies
Setting?	Convenient location for HCPs
Additional comments	Content of HCP training not defined. It was noted that training on BCTs would need to be tailored to this specific intervention.

*HCP* healthcare professional, *MDT* multidisciplinary team

## Discussion

We have identified eight relevant TDF domains influencing patient adherence to treatment (i.e., the mechanism of action), used these to define the content for an intervention comprising of 12 BCTs (i.e., the active ingredients) and used expert panels to determine how this intervention could be delivered in a research and clinical setting. To our knowledge, this is the first study to use this approach for a behaviour change intervention for patients with chronic respiratory disease. The content for our intervention has been developed in a systematic way and using psychological theories to identify 12 potentially effective and well-defined behavioural change techniques. It is hoped that by using this approach, we may increase the potential effectiveness of the intervention, its ability to be tested in other disease populations or be appraised as part of systematic reviews [11]. We used views of key stakeholders to determine how this intervention should be delivered, thereby, aiding its ability to be implemented into clinical practice [5]. If effective, this intervention has the potential to improve adherence and health outcomes for patients who suffer from a disease which can have daily symptoms which patients perceive as embarrassing [20], leads to frequent hospitalisations [24] and impairs quality of life [25].

The eight relevant TDF domains were the same for both patients and HCPs. A recent synthesis of factors affecting adherence reported similar findings to our eight relevant TDF domains for patients [26]. A single paper has used the TDF to identify what influenced patients’ behaviour towards haemodialysis care [27] and many studies have used the TDF to explore factors influencing HCP behaviours [28–31]. However, to our knowledge, no other studies have used the TDF to explore HCPs’ perceptions about their ability to influence adherence. Our data shows that HCPs lacked skills and confidence around changing patients’ adherence. HCPs identified that they felt they required training to help them manage adherence. Through the expert panels, these data were used to develop a plan for how HCPs could be trained to deliver this intervention. Further research is needed to outline the exact content of this training and develop the physical materials to facilitate it.

The results of our mapping of the TDF domains to BCTs are similar in both patients and HCPs, indicating that it was a rigorous process. Despite the potential for the TDF and BCTs to develop the content of interventions, few papers have used both frameworks in this way and all have focused on changing HCP behaviours [15, 16]. To our knowledge, this is the first paper to define the content of a behaviour change intervention by mapping the TDF to BCTs for both patients and HCPs.

The proposed intervention will be individualised for specific treatments and patients. This decision was taken prior to the expert panels taking place based on the strength and breadth of literature supporting this approach [4, 32, 33]. The HCP panel suggested that there would need to be an assessment visit to facilitate this. Several approaches could be used: (1) administering an assessment questionnaire to evaluate which TDF domains need to be targeted [34]; (2) by identifying responders and non-responders to particular BCTs or combinations of BCTs and using that to guide which BCTs to choose [35]. These approaches present considerable challenges for the design of a future randomised controlled trial. Future studies will focus on developing the supporting materials to allow the delivery of the 12 BCTs. As part of feasibility and pilot testing, we will explore the most appropriate methods of determining which BCTs to use and test the feasibility of these in different hospital settings.

The effectiveness of the proposed BCTs and plan for delivery has not yet been tested. Some of the 12 selected BCTs (information on behaviour and outcomes and cognitive restructuring) have been tested as part of other effective interventions [36, 37]. As these BCT were used with other intervention components, the extent to which they contributed to the effectiveness of these interventions is not known. Further, the optimum method of delivery for the included BCTs is not known. Given the variation in delivery of interventions [36, 37], it appears that the approach should be tailored to individual interventions and clinical settings.

There were a number of limitations. The TDF was not used to design the topic guide for the interviews or focus groups. Patients were recruited at the end of a related study on adherence; all patients coming to the end of the study were invited to participate until data saturation was reached. However, this may still have affected the study outcomes. The older compilation of BCTs was used as the more recent BCT taxonomy containing 93 BCTs was only published during the time our studies were conducted [17]. However, we attempted to consider any changes by cross-referencing the final list of included BCTs with the BCT taxonomy. Finally, whilst we obtained a broad range of views, it is difficult to know whether similar results would be found, particularly for delivery of the intervention, if this study was replicated in other healthcare settings with different infrastructure and systems in place. Snowball sampling of HCPs in stage 2 may have introduced bias; however, Northern Ireland has a small healthcare system and we recruited all of the key respiratory physicians, nurses and physiotherapists at each hospital site so this is unlikely to be a significant issue for this study.

## Conclusion

We identified 12 theory-derived BCTs that form the intervention content. Individually tailored content will be delivered to all patients over several face-to-face visits in secondary care. Future research should focus on developing physical materials to aid delivery of the proposed intervention prior to feasibility and pilot testing. If effective, this intervention may improve adherence and health outcomes for those with bronchiectasis in the future.

### Availability of supporting data

The datasets supporting the results of this article are included within the article (and its additional files).

## Additional files

Additional file 1:
**HCP topic guide and Theoretical Domains Framework used for analysis in Stage 1.** (DOCX 36 kb) Additional file 2: 
**BCT definitions, BCT mapping and reasons for exclusions of BCTs in Stage 2.** (DOCX 50 kb) Additional file 3: 
**Documents circulated to patient panel prior to attending.** (PDF 1208 kb) Additional file 4: 
**Documents circulated to HCP panel prior to attending.** (PDF 1314 kb) Additional file 5: 
**Materials used during patient expert panel.** (PDF 1477 kb) Additional file 6: 
**Materials used in HCP expert panel.** (PDF 1387 kb) Additional file 7: 
**Summary of patient expert panel findings.** (PDF 1049 kb) Additional file 8: 
**Summary of HCP expert panel findings.** (PDF 119 kb)

## Abbreviations

BCT: Behavioural change technique
CAN-BE: Change adherence to treatment in bronchiectasis
GP: General practitioner
HCP: Healthcare professionals
HSC: Health and social care trust
TDF: Theoretical domains framework.

## References

[1] Seitz AE, Olivier KN, Adjemian J, Holland SM, Prevots R (2012). Trends in bronchiectasis among medicare beneficiaries in the United States, 2000 to 2007. Chest.

[2] Pasteur MC, Bilton D, Hill AT (2010). Guideline for non-CF bronchiectasis. Thorax.

[3] McCullough A, Tunney M, Quittner A, Elborn J, Bradley J, Hughes C (2014). Treatment adherence and health outcomes in patients with bronchiectasis. BMC Pulm Med.

[4] McCullough AR, Ryan C, O’Neill B, Elborn JS, Bradley JM, Hughes CM (2014). Interventions for enhancing adherence to treatment in adults with chronic respiratory disease: a systematic review [abstract]. Am J Respir Crit Care Med.

[5] Medical Research Council. Developing and Evaluating Complex Interventions: New Guidance. 2008. http://www.mrc.ac.uk/documents/pdf/complex-interventions-guidance/

[6] National Institute for Health And Clinical Excellence (2007). Behaviour Change: The Principles for Effective Interventions.

[7] Michie S, Johnston M, Francis J, Hardeman W, Eccles M (2008). From theory to intervention: mapping theoretically derived behavioural determinants to behaviour change techniques. Appl Psychol.

[8] Apter AJ, Wang X, Bogen DK, Rand CS, McElligott S, Polsky D, et al. Problem solving to improve adherence and asthma outcomes in urban adults with moderate or severe asthma: a randomized controlled trial. J Allergy Clin Immunol. 2011;128:516–23. e1–5. 10.1016/j.jaci.2011.05.010 PMC3164914 21704360

[9] Armour C, Bosnic-Anticevich S, Brillant M, Burton D, Emmerton L, Krass I, et al. Pharmacy Asthma Care Program (PACP) improves outcomes for patients in the community. Thorax. 2007;62:496–502. 10.1136/thx.2006.064709 PMC2117224 17251316

[10] Gallefoss F, Bakke PS (1999). How does patient education and self-management among asthmatics and patients with chronic obstructive pulmonary disease affect medication?. Am J Respir Crit Care Med.

[11] Hoffmann TC, Glasziou PP, Barbour V, Macdonald H (2014). Better reporting of interventions: template for intervention description and replication (TIDieR) checklist and guide. Br Med J.

[12] Davidson K, Goldstein M, Kaplan R, Kaufman P, Knatterud G, Orleans C, et al. Evidence-based behavioural medicine: what is it and how do we achieve it? Ann Behav Med. 2003;26:161–71. 10.1207/S15324796ABM2603_01 14644692

[13] Michie S, Johnston M, Abraham C, Lawton R, Parker D (2005). Walker A, on behalf of the “Psychological Theory” group: making psychological theory useful for implementing evidence based practice: a consensus approach. Qual Saf Health Care.

[14] Abraham C, Michie S (2008). A taxonomy of behavior change techniques used in interventions. Health Psychol.

[15] Murphy K, O’Connor DA, Browning CJ, French SD, Michie S, Francis JJ, et al. Understanding diagnosis and management of dementia and guideline implementation in general practice: a qualitative study using the theoretical domains framework. Implement Sci. 2014;9:31. 10.1186/1748-5908-9-31 PMC4015883 24581339

[16] French SD, Green SE, O’Connor DA, McKenzie JE, Francis JJ, Michie S, et al. Developing theory-informed behaviour change interventions to implement evidence into practice: a systematic approach using the Theoretical Domains Framework. Implement Sci. 2012;7:38. 10.1186/1748-5908-7-38 PMC3443064 22531013

[17] Michie S, Richardson M, Johnston M, Abraham C, Francis J, Hardeman W, et al. The behavior change technique taxonomy (v1) of 93 hierarchically clustered techniques: building an international consensus for the reporting of behavior change interventions. Ann Behav Med. 2013;46:81–95. 10.1007/s12160-013-9486-6 23512568

[18] Cane J, O’Connor D, Michie S (2012). Validation of the theoretical domains framework for use in behaviour change and implementation research. Implement Sci.

[19] Kolehmainen N, Francis JJ (2012). Specifying content and mechanisms of change in interventions to change professionals’ practice: an illustration from the Good Goals study in occupational therapy. Implement Sci.

[20] McCullough A, Tunney M, Elborn J, Bradley J, Hughes C. “All illness is personal to that individual”: a qualitative study of patients’ perspectives on treatment adherence in bronchiectasis. Heal Expect. 2014. doi:10.1111/hex.12217. 10.1111/hex.12217 PMC5810642 24948008

[21] King N, Horrocks C (2010). Interviews in Qualitative Research.

[22] Francis JJ, Stockton C, Eccles MP, Johnston M, Cuthbertson BH, Grimshaw JM, et al. Evidence-based selection of theories for designing behaviour change interventions: using methods based on theoretical construct domains to understand clinicians’ blood transfusion behaviour. Br J Health Psychol. 2009;14:625–46. 10.1348/135910708X397025 19159506

[23] McCullough A, Tunney MM, Quittner AL, Elborn JS, Bradley JM, Hughes CM. Treatment adherence and health outcomes in patients with bronchiectasis. BMC Pulm Med. 2014;14:107. 10.1186/1471-2466-14-107 PMC4090650 24980161

[24] Seitz AE, Olivier KN, Steiner CA, Montes de Oca R, Holland SM, Prevots DR (2010). Trends and burden of bronchiectasis-associated hospitalizations in the United States, 1993-2006. Chest.

[25] Quittner AL, Marciel KK, Salathe MA, O’Donnell AE, Gotfried MH, Ilowite JS, et al. A preliminary quality of life questionnaire-bronchiectasis: a patient-reported outcome measure for bronchiectasis. Chest. 2014;146:437. 10.1378/chest.13-1891 24626872

[26] Jackson C, Eliasson L, Barber N, Weinman J (2014). Applying COM-B to medication adherence: a suggested framework for research and interventions. Eur Heal Psychol.

[27] Glidewell L, Boocock S, Pine K, Campbell R, Hackett J, Gill S, et al. Using behavioural theories to optimise shared haemodialysis care: a qualitative intervention development study of patient and professional experience. Implement Sci. 2013;8:118. 10.1186/1748-5908-8-118 PMC3851734 24098920

[28] Duncan EM, Francis JJ, Johnston M, Davey P, Maxwell S, McKay GA, et al. Learning curves, taking instructions, and patient safety: using a theoretical domains framework in an interview study to investigate prescribing errors among trainee doctors. Implement Sci. 2012;7:86. 10.1186/1748-5908-7-86 PMC3546877 22967756

[29] McSherry LA, Dombrowski SU, Francis JJ, Murphy J, Martin CM, O’Leary JJ, et al. “It”s a can of worms’: understanding primary care practitioners' behaviours in relation to HPV using the Theoretical Domains Framework. Implement Sci. 2012;7:73. 10.1186/1748-5908-7-73 PMC3523072 22862968

[30] Bussières AE, Patey AM, Francis JJ, Sales AE, Grimshaw JM, Brouwers M, et al. Identifying factors likely to influence compliance with diagnostic imaging guideline recommendations for spine disorders among chiropractors in North America: a focus group study using the Theoretical Domains Framework. Implement Sci. 2012;7:82. 10.1186/1748-5908-7-82 PMC3444898 22938135

[31] Islam R, Tinmouth AT, Francis JJ, Brehaut JC, Born J, Stockton C, et al. A cross-country comparison of intensive care physicians’ beliefs about their transfusion behaviour: a qualitative study using the Theoretical Domains Framework. Implement Sci. 2012;7:93. 10.1186/1748-5908-7-93 PMC3527303 22999460

[32] Zullig LL, Peterson ED, Bosworth HB (2013). Ingredients of successful interventions to improve medication adherence. JAMA.

[33] Haynes RB, Ackloo E, Sahota N, McDonald HP, Yao X. Interventions for enhancing medication adherence. Cochrane Database of Systematic Reviews 2008; 2: CD000011. 10.1002/14651858.CD000011.pub3 18425859

[34] Michie S, Atkins L, West R (2014). The behaviour change wheel: a guide to designing interventions.

[35] Winett RA, Davy BM, Savla J, Marinik EL, Winett SG, Baugh ME, et al. Using response variation to develop more effective, personalized behavioral medicine?: evidence from the Resist Diabetes study. Transl Behav Med. 2014;4:333–8. 10.1007/s13142-014-0263-2 PMC4167893 25264472

[36] Wilson SR, Strub P, Buist AS, Knowles SB, Lavori PW, Lapidus J, et al. Shared treatment decision making improves adherence and outcomes in poorly controlled asthma. Am J Respir Crit Care Med. 2010;181:566–77. 10.1164/rccm.200906-0907OC PMC2841026 20019345

[37] Trupp RJ, Corwin EJ, Ahijevych KL, Nygren T (2011). The impact of educational message framing on adherence to continuous positive airway pressure therapy. Behav Sleep Med.

