# Supplementary material for: Defining the content and delivery of an intervention to Change AdhereNce to treatment in BonchiEctasis (CAN-BE): a qualitative approach incorporating the Theoretical Domains Framework, behavioural change techniques and stakeholder expert panels
Source: BMC Health Serv Res. 2015 Aug 22;15:342. doi: 10.1186/s12913-015-1004-z (PMC4546345; doi:10.1186/s12913-015-1004-z)
Supplement: Additional file 1: — HCP topic guide and Theoretical Domains Framework used for analysis in Stage 1. (DOCX 36 kb) [file 12913_2015_1004_MOESM1_ESM.docx]

**Additional file 1 –Topic guide and copies of TDF used**

**Study title: Development of a bronchiectasis-specific intervention focusing on adherence to treatment in bronchiectasis**

**Topic guide**

**Introduction**

Thank you all for coming along to today’s focus group discussion. The purpose of this discussion is to explore healthcare professionals’ views on adherence to treatment in patients with bronchiectasis. This data will then be used together with our patient data collected previously and the literature on adherence interventions in respiratory disease to develop an intervention focusing on adherence to treatment in patients with bronchiectasis. All of your answers will be treated in confidence and you will not be identified in any of the subsequent reports. We would ask that you do not name any specific patients during this discussion and ask that you maintain the confidentiality of this discussion by not sharing it with others outside of this focus group. The discussion will last a maximum of two hours and will be audio-recorded. I will have a list of questions which I will use to guide the discussion. Cris and I may also take notes during the discussion. Cris will note some of the key points from the discussion on a flip chart/white board for us to discuss further. Are you happy to proceed?

**Introductory question**

1. Can you each introduce yourself, describe your clinical background and indicate your general role in the management of patients with bronchiectasis?
2. Describe the services that you and your clinical department provide for patients with bronchiectasis.

**Key questions**

1. What is your understanding of adherence to treatment for patients with bronchiectasis?
2. What do you think the barriers are for patients to adhere to treatment?
3. What do you think enables or motivates patients to adhere to treatment?
4. What is your current role in the management of adherence to treatment for patients with bronchiectasis?
5. What are the barriers to you conducting this role?
6. What enables you to carry out your role in promoting adherence?
7. Which barriers could be overcome and which motivators to adherence could be enhanced in the current clinical environment?
8. Who could do this?
9. What could they do?
10. How could they do it?
11. Which patients would you target with these adherence strategies?
12. How could you measure the effect of these changes on patient outcomes?
13. We’ve identified several ideas for improving adherence, how do you think I should prioritise and take these ideas forward?

**Conclusion**

1. Have I missed anything with the questions I have asked?
2. Do you have anything further you’d like to raise?

**Thanks for your time!**

**Adaption of TDF for use with patient data**

The 12 domain TDF was developed for use with healthcare professionals and the domain content descriptions were written for this purpose (Table 1). These descriptions could not be applied directly to patient data [13]. To overcome this, we used patient-focused domain descriptions described by Glidewell *et al*. [25] (Table 1). However, Glidewell *et al*. [25] did not have domain content descriptions for two of the TDF domains (Motivation and Nature of Behaviours). We generated domain content descriptions for these domains by adapting the descriptions from the HCP version of the TDF (Table 2) [13].

**Table 1. 12 domain TDF used to analyse patient data** [13,25]

|  | **Domain labels** [13] | **Domain content** | **Domain constructs** [13] |
| --- | --- | --- | --- |
| 1 | Knowledge | An awareness of the existence of something [25]. | Knowledge  Procedural knowledge  Knowledge about condition/scientific rationale  Schemas, mindsets and illness representations |
| 2 | Skills | An ability or proficiency acquired through practice [25]. | Skills  Interpersonal skills  Competence/ability/skill assessment  Practice/skill development  Coping strategies |
| 3 | Social/Professional role & identity | A coherent set of behaviours and displayed personal qualities of an individual in a social or work setting [25]. | Identity  Professional identity/boundaries/role  Group/social identity  Social/group norms  Alienation/organisational commitment |
| 4 | Beliefs about capabilities | Acceptance of the truth, reality, or validity about an ability, talent or facility that a person can put to constructive use [25]. | Self efficacy  Control of behaviour an material and social environment  Perceived competence  Self-confidence/ professional confidence  Empowerment  Self-esteem  Perceived behavioural control  Optimism/pessimism |
| 5 | Beliefs about consequences | Acceptance of the truth, reality or validity about outcomes of a behaviour in a given situation [25]. | Outcome expectancies  Anticipated regret  Appraisal/evaluation/consequences  Incentives/rewards  Beliefs  Unrealistic optimism  Salient events/sensitisation/critical incidents  Characteristics of outcome expectancies – physical, social, emotional |
| 6 | Motivation & goals | The relative priority given to one issue compared to other demands [Adapted from 13]. Mental representations of outcome or end states that an individual wants to achieve (Goals) [25]. | Intention  Stability of intention/certainty of intention  Goals (autonomous, controlled)  Goal target/setting  Goal propriety  Intrinsic motivation  Commitment  Distal and proximal goals  Transtheoretical model and stages of change |
| 7 | Memory, attention & decision processes | The ability to retain information, focus selectively on aspects of the environment and choose between two or more alternatives [25]. | Memory  Attention  Attention control  Decision making |
| 8 | Environmental context/resources | Any circumstance of a person’s situation or environment that discourages or encourages the development of skills and abilities, independence, social competence, and adaptive behaviour [25]. | Resources/material resources (availability and management)  Environmental stressors  Person x environmental interaction  Knowledge of task/environment |
| 9 | Social influences | Those interpersonal processes that can cause individuals to change their thoughts, feelings, or behaviour [25]. | Social support  Social/group norms  Organisational development  Leadership  Team working  Group conformity  Organisational climate/culture  Social pressure  Power/hierarchy  Professional boundaries/roles  Management commitment  Supervision  Inter-group conflict  Champions  Social comparison  Identity  Group/social identity  Organisation commitment/alienation  Feedback  Negotiation  Conflict-competing demands  Conflicting roles  Change management  Crew resource management  Social support: personal/professional/organisational, intra/interpersonal, socity/community  Social/group norms: subjective, descriptive, injunctive norms  Learning and modelling |
| 10 | Emotion | A complex reaction pattern, involving experimental, behavioural and physiological elements, by which the individual attempts to deal with a personally significant matter or event [25]. | Affect  Stress  Anticipated regret  Fear  Burn-out  Cognitive overload/tiredness  Threat  Positive/negative affect  Anxiety/depression |
| 11 | Behavioural regulation | Anything aimed at managing or changing objectively observed or measured actions [25]. | Goal/target setting  Implementation intention  Action planning  Self-monitoring  Goal priority  Generating alternatives  Feedback  Moderators of intention-behaviour gap  Project management  Barriers and facilitators |
| 12 | Nature of the behaviours | Some new behaviours are very similar to current behaviour and so are easier to implement than new behaviours that require a dramatic change in ways of life [Adapted from 13]. | Routine/automatic/habit  Breaking habit  Direct experience/past behaviour  Representation of tasks  Stages of change model |

**Table 2. 12 domain TDF used to analyse HCP data** [13]

|  | **Domain labels** | **Domain content** | **Domain constructs** |
| --- | --- | --- | --- |
| 1 | Knowledge | Knowledge of the field (i.e. whether there is adequate evidence) and individuals’ knowledge of the evidence or of a guideline. | Knowledge  Procedural knowledge  Knowledge about condition/scientific rationale  Schemas, mindsets and illness representations |
| 2 | Skills | Covers the possibility that new skills would be required by the staff who are required to implement a new procedure. | Skills  Interpersonal skills  Competence/ability/skill assessment  Practice/skill development  Coping strategies |
| 3 | Social/Professional role & identity | The clinical thinking and norms of a particular profession. | Identity  Professional identity/boundaries/role  Group/social identity  Social/group norms  Alienation/organisational commitment |
| 4 | Beliefs about capabilities | How confident clinicians are that they could change their practice effectively. | Self efficacy  Control of behaviour an material and social environment  Perceived competence  Self-confidence/ professional confidence  Empowerment  Self-esteem  Perceived behavioural control  Optimism/pessimism |
| 5 | Beliefs about consequences | Often regarded as core to clinical reasoning, this domain covers the perceived benefits and harms of a clinical action. In some contexts it can also include consequences for the clinician such as workload, pay, career progression, or for the hospital or health service. | Outcome expectancies  Anticipated regret  Appraisal/evaluation/consequences  Incentives/rewards  Beliefs  Unrealistic optimism  Salient events/sensitisation/critical incidents  Characteristics of outcome expectancies – physical, social, emotional |
| 6 | Motivation & goals | The relative priority that is given to one clinical issue, compared with other demands. | Intention  Stability of intention/certainty of intention  Goals (autonomous, controlled)  Goal target/setting  Goal propriety  Intrinsic motivation  Commitment  Distal and proximal goals  Transtheoretical model and stages of change |
| 7 | Memory, attention & decision processes | The level of attention that is needed to perform the key clinical action (ie is forgetting likely to be a problem) and the processes by which clinical decisions are made by individuals and teams. | Memory  Attention  Attention control  Decision making |
| 8 | Environmental context/resources | Includes the physical (including financial) issues that may limit change, including staffing levels and time as well as equipment or space. | Resources/material resources (availability and management)  Environmental stressors  Person x environmental interaction  Knowledge of task/environment |
| 9 | Social influences | The influence of other individuals or groups on clinical practice, for example, patients, patients’ families, pressure groups. | Social support  Social/group norms  Organisational development  Leadership  Team working  Group conformity  Organisational climate/culture  Social pressure  Power/hierarchy  Professional boundaries/roles  Management commitment  Supervision  Inter-group conflict  Champions  Social comparison  Identity  Group/social identity  Organisation commitment/alienation  Feedback  Negotiation  Conflict-competing demands  Conflicting roles  Change management  Crew resource management  Social support: personal/professional/organisational, intra/interpersonal, socity/community  Social/group norms: subjective, descriptive, injunctive norms  Learning and modelling |
| 10 | Emotion | Includes issues such as work stress, patient anxiety and other emotional factors that may help or hinder the uptake of new approaches to care. | Affect  Stress  Anticipated regret  Fear  Burn-out  Cognitive overload/tiredness  Threat  Positive/negative affect  Anxiety/depression |
| 11 | Behavioural regulation | Includes the ‘how’ of changing clinical practice: what are the practical strategies that would facilitate or hinder uptake of a new practice. | Goal/target setting  Implementation intention  Action planning  Self-monitoring  Goal priority  Generating alternatives  Feedback  Moderators of intention-behaviour gap  Project management  Barriers and facilitators |
| 12 | Nature of the behaviours | Some new practices are very similar to current practice and so are easier to implement than new practices that require a dramatic change in ways of working. | Routine/automatic/habit  Breaking habit  Direct experience/past behaviour  Representation of tasks  Stages of change model |
