# Supplementary material for: Defining the content and delivery of an intervention to Change AdhereNce to treatment in BonchiEctasis (CAN-BE): a qualitative approach incorporating the Theoretical Domains Framework, behavioural change techniques and stakeholder expert panels
Source: BMC Health Serv Res. 2015 Aug 22;15:342. doi: 10.1186/s12913-015-1004-z (PMC4546345; doi:10.1186/s12913-015-1004-z)
Supplement: Additional file 2: — BCT definitions, BCT mapping and reasons for exclusions of BCTs in Stage 2. (DOCX 50 kb) [file 12913_2015_1004_MOESM2_ESM.docx]

**Additional File 2 – BCT definitions, BCT mapping and reasons for BCT exclusions**

**Table 1. List of 35 BCTs and their definitions** [13]

| **BCT** | **Definition** |
| --- | --- |
| Goal/target specified: behaviour or outcome | Set or agree on a goal defined in terms of the behaviour to be achieved. Set or agree on a goal defined in terms of a positive outcome of wanted behaviour |
| Monitoring | Record specified behaviour. |
| Self-monitoring | Record own specified behaviour. |
| Contract | Of agreed performance of target behaviour with at least one other, written and signed. |
| Rewards; incentives including self-evaluation | Contingent valued consequence, i.e. if and only if behaviour is performed (inc. social approval, exc. general non-contingent encouragement or approval). |
| Graded task, starting with easy tasks | Set easy tasks to perform, making them increasingly difficult until target behaviour performed. |
| Increasing skills: problem solving, decision making, goal setting | No definition provided. |
| Stress management | Behaviours undertaken to reduce stressors or impact of stressors. |
| Coping skills | Behaviours undertaken to avoid or reduce stressors. |
| Rehearsal of relevant skills | Behavioural rehearsal: perform behaviour (repeatedly). Mental rehearsal: imagine performing the behaviour repeatedly. |
| Role play | Perform behaviour in simulated situation. |
| Planning, implementation | Identify component parts of behaviour and make plan to execute each one or consider when and/or where a behaviour will be performed, i.e. schedule behaviours. |
| Prompts, triggers, cues | Stimulus that elicits behaviour (inc. telephone calls or postal reminders designed to prompt the behaviour). |
| Environmental changes e.g. objects to facilitate behaviour | Change the environment in order to facilitate the target behaviour (other than prompts, rewards, and punishments e.g. choice of food provided). |
| Social processes of encouragement, pressure, support | Social support (emotional): others listen, provide empathy and give generalised positive feedback. |
| Persuasive communication | Credible source presents arguments in favour of the behaviour. Note, there must be evidence of presentation of arguments; general pro-behaviour communication does not count. |
| Information regarding behaviour, outcome | Behavioural information: provide information about antecedents or consequences of the behaviour, or connections between them, or behaviour change techniques. |
| Personalised message | Tailor techniques or messages from others to individual’s resources and context (includes stages of change-based information; doesn’t include personal plans and feedback). |
| Modelling/ demonstration of behaviour by others | Observe the behaviour of others. |
| Homework | Set homework tasks. |
| Personal experiments, data collection (other than self-monitoring of behaviour) | No definition provided. |
| Experiential; tasks to gain experiences to change motivation | No definition provided. |
| Feedback | Of monitored (including self-monitored behaviour). |
| Self-talk | Planned self-statements (aloud or silent) to implement behaviour change techniques (incl mental rehersal). |
| Use of imagery | Use planned images (visual, motor, sensory) to implement behaviour change techniques (inc. mental rehearsal). |
| Perform behaviour in different settings | No definition provided |
| Shaping of behaviour | Build up behaviour by initially reinforcing behaviour closest to required behaviour and systematically altering behaviour required to achieve contingent reinforcement. |
| Motivational interviewing | Elicit self-motivating statements and evaluation of own behaviour to reduce resistance to change. |
| Relapse prevention | Identify situations that increase the likelihood of the behaviour not being performed and apply coping strategies to those situations. |
| Cognitive restructuring | Changing cognitions about causes and consequences of behaviour. |
| Relaxation | Systematic instruction in physical and cognitive strategies to reduce sympathetic arousal, and to increase muscle relaxation and a feeling of calm. |
| Desensitisation | Exposure to threatening experiences. |
| Problem solving | No definition provided. |
| Time management | Action planning applied to the perceived problem of shortage of time. |
| Identify/prepare for difficult situation/ problems | No definition provided. |

**Table 2. Mapping of relevant patient TDF domains to BCTs^a^ (Stage 2)**

|  |  | **TDF domains** | | | | | | | |
| --- | --- | --- | --- | --- | --- | --- | --- | --- | --- |
| **Rank** | **BCTs** [13] | **Knowledge** | **Skills** | **Capability** | **Consequences** | **Motivation** | **Social** | **Behavioural regulation** | **Nature of behaviours** |
| 1 | Cognitive restructuring |  |  |  |  |  |  |  |  |
| 2 | Graded task, starting with easy tasks |  |  |  |  |  |  |  |  |
| 3 | Information regarding behaviour, outcome |  |  |  |  |  |  |  |  |
| 4 | Persuasive communication |  |  |  |  |  |  |  |  |
| 5 | Shaping of behaviour |  |  |  |  |  |  |  |  |
| 6 | Identify/prepare for difficult situation/ problems |  |  |  |  |  |  |  |  |
| 7 | Self-monitoring |  |  |  |  |  |  |  |  |
| 8 | Role play |  |  |  |  |  |  |  |  |
| 9 | Motivational interviewing |  |  |  |  |  |  |  |  |
| 10 | Time management |  |  |  |  |  |  |  |  |
| 11 | Problem solving |  |  |  |  |  |  |  |  |
| 12 | Goal/target specified: behaviour or outcome |  |  |  |  |  |  |  |  |
| 13 | Rewards; incentives including self-evaluation |  |  |  |  |  |  |  |  |
| 14 | Modelling/ demonstration of behaviour by others |  |  |  |  |  |  |  |  |
| 15 | Relapse prevention |  |  |  |  |  |  |  |  |
| 16 | Monitoring |  |  |  |  |  |  |  |  |
| 17 | Feedback |  |  |  |  |  |  |  |  |
| 18 | Rehearsal of relevant skills |  |  |  |  |  |  |  |  |
| 19 | Social processes of encouragement, pressure, support |  |  |  |  |  |  |  |  |
| 20 | Personalised message |  |  |  |  |  |  |  |  |
| 21 | Experiential; tasks to gain experiences to change motivation |  |  |  |  |  |  |  |  |
| 22 | Increasing skills: problem solving, decision making, goal setting |  |  |  |  |  |  |  |  |
| 23 | Contract |  |  |  |  |  |  |  |  |
| 24 | Homework |  |  |  |  |  |  |  |  |
| 25 | Personal experiments, data collection (other than self-monitoring of behaviour) |  |  |  |  |  |  |  |  |
| 26 | Planning, implementation |  |  |  |  |  |  |  |  |
| 27 | Coping skills |  |  |  |  |  |  |  |  |
| 28 | Use of imagery |  |  |  |  |  |  |  |  |
| 29 | Self-talk |  |  |  |  |  |  |  |  |
| 30 | Desensitisation |  |  |  |  |  |  |  |  |
| 31 | Stress management |  |  |  |  |  |  |  |  |
| 32 | Prompts, triggers, cues |  |  |  |  |  |  |  |  |
| 33 | Environmental changes e.g. objects to facilitate behaviour |  |  |  |  |  |  |  |  |
| 34 | Relaxation |  |  |  |  |  |  |  |  |
| 35 | Perform behaviour in different settings |  |  |  |  |  |  |  |  |

^a^BCTs ranked by the number of TDF domains for which there was ‘agreed use’ or ‘agreed use’ plus ‘disagreement.’

**Coding key:**

Agreed use = Two or more raters with a 2 or 3 except if the third rater has is a zero

Agreed non-use = Two or more zeros with any other rating

Disagreement = One rating of zero and two raters with a 2 or 3

Uncertain = all other cells in the matrix

**Table 3. Mapping of relevant HCP TDF domains to BCTs^a^ (Stage 2)**

|  |  | **TDF domains** | | | | | | | |
| --- | --- | --- | --- | --- | --- | --- | --- | --- | --- |
| **Rank** | **BCTs** [13] | **Knowledge** | **Skills** | **Capability** | **Consequences** | **Motivation** | **Social** | **Behaviour regulation** | **Nature of behaviour** |
| 1 | Monitoring |  |  |  |  |  |  |  |  |
| 2 | Self-monitoring |  |  |  |  |  |  |  |  |
| 3 | Feedback |  |  |  |  |  |  |  |  |
| 4 | Time management |  |  |  |  |  |  |  |  |
| 5 | Identify/prepare for difficult situation/ problems |  |  |  |  |  |  |  |  |
| 6 | Rewards; incentives including self-evaluation |  |  |  |  |  |  |  |  |
| 7 | Persuasive communication |  |  |  |  |  |  |  |  |
| 8 | Goal/target specified: behaviour or outcome |  |  |  |  |  |  |  |  |
| 9 | Contract |  |  |  |  |  |  |  |  |
| 10 | Graded task, starting with easy tasks |  |  |  |  |  |  |  |  |
| 11 | Increasing skills: problem solving, decision making, goal setting |  |  |  |  |  |  |  |  |
| 12 | Information regarding behaviour, outcome |  |  |  |  |  |  |  |  |
| 13 | Modelling/ demonstration of behaviour by others |  |  |  |  |  |  |  |  |
| 14 | Self-talk |  |  |  |  |  |  |  |  |
| 15 | Relapse prevention |  |  |  |  |  |  |  |  |
| 16 | Social processes of encouragement, pressure, support |  |  |  |  |  |  |  |  |
| 17 | Cognitive restructuring |  |  |  |  |  |  |  |  |
| 18 | Motivational interviewing |  |  |  |  |  |  |  |  |
| 19 | Perform behaviour in different settings |  |  |  |  |  |  |  |  |
| 20 | Problem solving |  |  |  |  |  |  |  |  |
| 21 | Rehearsal of relevant skills |  |  |  |  |  |  |  |  |
| 22 | Personalised message |  |  |  |  |  |  |  |  |
| 23 | Coping skills |  |  |  |  |  |  |  |  |
| 24 | Role play |  |  |  |  |  |  |  |  |
| 25 | Homework |  |  |  |  |  |  |  |  |
| 26 | Personal experiments, data collection (other than self-monitoring of behaviour) |  |  |  |  |  |  |  |  |
| 27 | Experiential; tasks to gain experiences to change motivation |  |  |  |  |  |  |  |  |
| 28 | Prompts, triggers, cues |  |  |  |  |  |  |  |  |
| 29 | Planning, implementation |  |  |  |  |  |  |  |  |
| 30 | Stress management |  |  |  |  |  |  |  |  |
| 31 | Environmental changes e.g. objects to facilitate behaviour |  |  |  |  |  |  |  |  |
| 32 | Use of imagery |  |  |  |  |  |  |  |  |
| 33 | Shaping of behaviour |  |  |  |  |  |  |  |  |
| 34 | Relaxation |  |  |  |  |  |  |  |  |
| 35 | Desensitisation |  |  |  |  |  |  |  |  |

^a^BCTs ranked by the number of TDF domains for which there was ‘agreed use’ or ‘agreed use’ plus ‘disagreement.’

**Coding key:**

Agreed use = Two or more raters with a 2 or 3 except if the third rater has is a zero

Agreed non-use = Two or more zeros with any other rating

Disagreement = One rating of zero and two raters with a 2 or 3

Uncertain = all other cells in the matrix

**Table 4. Reasons for exclusion of patient BCTs**

|  | **BCT** | **Reason for exclusion** |
| --- | --- | --- |
| 6 | Identify/ prepare for difficult /situations problems. | Not included in BCT Taxonomy in this form. Included under Problem solving. Effective systematic review studies used problem solving strategies; therefore, problem solving included instead. |
| 13 | Rewards; incentives including self-evaluation | Reward approximation used under Shaping of behaviour in BCT taxonomy. Reward was not tested in systematic review and so its effectiveness is not known. |
| 14 | Modelling/ demonstration of behaviour by others | Overlaps with Role play in BCT taxonomy. Not tested in studies in systematic review so its effectiveness is not known. |
| 15 | Relapse prevention | Already included under problem solving. Effective systematic review studies used these problem solving strategies. |
| 18 | Rehearsal of relevant skills | Already included under Role play BCT. Systematic review referred to teaching inhaler technique skills but not enough detail to know if this involved rehearsal. |
| 19 | Social processes of encouragement, pressure, support | Grouped with Motivational interviewing in BCT taxonomy under Social support. No specific studies in systematic review to support or refute inclusion. |
| 20 | Personalised message | Removed from BCT taxonomy. No data in systematic review to support or refute inclusion. |
| 21 | Experiential; tasks to gain experiences to change motivation | Removed from BCT taxonomy. No data in systematic review to support or refute inclusion. |
| 22 | Increasing skills: problem solving, decision making, goal setting | Removed from BCT taxonomy. No data in systematic review to support or refute inclusion. |
| 23 | Contract | No data in systematic review to support or refute inclusion. |
| 24 | Homework | Removed from BCT taxonomy. No data in systematic review to support or refute inclusion. |

**Table 5. Reasons for exclusion of healthcare professional BCTs**

|  | **BCTs** | **Reason for exclusion** |
| --- | --- | --- |
| 5 | Identify/ prepare for difficult /situations problems | Not included in BCT Taxonomy in this form. Included under Problem solving. |
| 13 | Modelling/ demonstration of behaviour by others | Overlaps with Role play in BCT taxonomy. |
| 18 | Motivational interviewing | Overlaps with ‘Social processes of encouragement, pressure, support’ BCT, which is grouped with Motivation Interviewing in BCT taxonomy under ‘Social support’. |
| 19 | Perform behaviour in different settings | Overlaps with Role play in BCT taxonomy. |
| 21 | Rehearsal of relevant skills | Overlaps with Role play in BCT taxonomy. |
| 22 | Personalised message | Removed from BCT Taxonomy. |
| 23 | Coping skills | Included under Problem-solving. Removed from BCT Taxonomy. |
| 25 | Homework | Removed from BCT taxonomy. |
| 26 | Personal experiments, data collection (other than self-monitoring of behaviour) | Removed from BCT taxonomy. Overlaps with Monitoring, Self-monitoring and Feedback. |
| 27 | Experiential; tasks to gain experiences to change motivation | Removed from BCT taxonomy. Overlaps with Monitoring, Self-monitoring and Feedback. |
