# Supplementary material for: Defining the content and delivery of an intervention to Change AdhereNce to treatment in BonchiEctasis (CAN-BE): a qualitative approach incorporating the Theoretical Domains Framework, behavioural change techniques and stakeholder expert panels
Source: BMC Health Serv Res. 2015 Aug 22;15:342. doi: 10.1186/s12913-015-1004-z (PMC4546345; doi:10.1186/s12913-015-1004-z)
Supplement: Additional file 3: — Documents circulated to patient panel prior to attending. (PDF 1208 kb) [file 12913_2015_1004_MOESM3_ESM.pdf]

Ulster University and Queen's University Logos here

Bronchiectasis meeting

Monday 28th April 2014

Room N09/10, McClay Research Centre, School of Pharmacy, Queen's University Belfast

What is the purpose of the meeting?

Bronchiectasis is a chronic lung condition which affects many people in Northern Ireland. It can make people feel short of breath, have a regular cough and produce more phlegm. Patients with bronchiectasis need treatments including inhalers, nebulisers and physiotherapy. From other research studies we have carried out, we know that patients with bronchiectasis sometimes find it hard to do all of their treatments. So, we have put together a plan that we think might be able to improve this for patients. It is important that patients are involved in developing this plan. The aim of this meeting is to get your views on this plan and to help us work out how we might be able to use this in everyday life.

What research have we completed so far?

We have completed four research studies.

The first study included 75 patients with bronchiectasis from across Northern Ireland. We asked them about whether they took their treatments as prescribed over the course of one year.

The **key results** from this study were:

- Patients said that they did not do all of their treatments as prescribed.
- Patients who didn't take their inhaled antibiotics (Colomycin® or tobramycin) as prescribed had more chest infections than those who did.

- Patients who believed they needed airway clearance were more likely to do this treatment. Patients who were concerned about side effects of medications were less likely to take them.

The second research study included 16 patients with bronchiectasis from across Northern Ireland. They took part in an interview with a researcher at their hospital or in their own home. Patients were asked about their views on their treatments for bronchiectasis and what difficulties they had doing their treatments.

The **key results** from this study were:

- Patients had difficulties completing all of their treatments for bronchiectasis.
- Patients said that the following things influenced whether they did a treatment:
    - Their knowledge about disease and treatment.
    - Their ability to be able to do that treatment.
    - Whether they believed in their own ability to do that treatment.
    - Whether they believed they needed to do that treatment or not.
    - Their motivation to do that treatment.
    - Having support from healthcare professionals, family members and friends.
    - Having practical ways of fitting treatments into their day, such as setting reminders on their phone.
    - Having a routine for treatment.
- Patients also gave us ideas of it could be made easier for them to do all of their treatments. They thought that:
    - Patients need more education on bronchiectasis and its treatment.
    - Patients need to know about the negative consequences of not doing treatments.
    - Patients should get into a routine by planning treatment into their day.
    - Patients could use reminders to help them to remember to do treatments.
    - Patients could use family members to help them with treatment.
    - Easier access to some healthcare professionals was needed.

The third study included 46 healthcare professionals (doctors, nurses, physiotherapists, pharmacists and psychologists) from across Northern Ireland. They took part in interviews with researchers. They were asked about what could be done to make it easier for patients to their treatments for bronchiectasis.

The **key results** from this study were:

- Patients need more education on bronchiectasis and its treatment.
- Patients need information about the negative consequences of not doing treatments.
- Using self-management plans helps patients to take their treatments.
- Better communication is needed between patients and healthcare professionals.

In the fourth study, we looked at what other researchers have done to try and make it easier for patients with all types of chest problems to do their treatments.

The **key results** from this were:

- We found 51 research studies that looked at ways of making it easier for patients to take treatments.
- Studies that were effective used a number of ways to change patients' behaviour.
- Most studies educated patients about their disease and treatment. However, they also used practical ways of changing behaviour such as, setting goals with patients, planning how to deal with changes in treatment and solving problems that patients faced.

How have we used the information from all of these studies?

We have combined the information that we have from all four studies. We have used this information to develop a plan that we think could make it easier for patients to do their treatments.

What will happen during the meeting?

Please see below a programme for the meeting. The main focus of the meeting will be to present the plan that we think could make it easier for patients to do their treatments. We will ask you to think about how we might be able to use this in everyday life. You will be asked to take part in a discussion with other patients with bronchiectasis. Following the meeting, we will use the information you give us to develop the plan further. We will send you an updated version of the plan and we will contact you to get your opinion on this.

Programme for the meeting

9.30-10.00am	**Refreshments and consent**
10.00-10.20am	Welcome and introduction
10.20-11.00am	Presentation: *How did we create our plan to make it easier for patients to do their treatments?*
11.00-11.15am	**Coffee**
11.15-12.15pm	Group discussion: *How can we use this plan in everyday life?*
12.15-12.45pm	Summary of discussion
12.45-1.00pm	**Close**
