# Supplementary material for: Defining the content and delivery of an intervention to Change AdhereNce to treatment in BonchiEctasis (CAN-BE): a qualitative approach incorporating the Theoretical Domains Framework, behavioural change techniques and stakeholder expert panels
Source: BMC Health Serv Res. 2015 Aug 22;15:342. doi: 10.1186/s12913-015-1004-z (PMC4546345; doi:10.1186/s12913-015-1004-z)
Supplement: Additional file 4: — Documents circulated to HCP panel prior to attending. (PDF 1314 kb) [file 12913_2015_1004_MOESM4_ESM.pdf]

Queen's University Belfast and Ulster University Logo here

EXPERT PANEL DOCUMENT

Study title: Development of a bronchiectasis-specific adherence intervention

Investigators: Dr Amanda McCullough, Dr Cristin Ryan, Dr Brenda O'Neill, Prof. Stuart Elborn, Prof. Judy Bradley and Prof. Carmel Hughes

CONTENTS

Background	3
Approach to intervention development	4
Research outputs	9
References	10
Directions to meeting	13

BACKGROUND

Bronchiectasis is an under-researched respiratory disease, which is characterised by chronic, excessive sputum production and recurrent infection[1]. Aetiologies include previous respiratory tract infection and immune defects[2]. Until recently, it had been considered an 'orphan disease'[3] but is now recognised as a disease of 'growing importance' worldwide[4]; despite this, the evidence base is still poor. Globally, the prevalence of bronchiectasis is undetermined[5]. It has been estimated that 110,000 people in the United States have bronchiectasis with an associated healthcare cost of US $630 million[6]. In England and Wales, a recent study showed that approximately 1000 patients die from bronchiectasis annually, with a yearly increase of 3% from 2001[7]; however this may be an under-estimation[7].

Little research exists for treatments in bronchiectasis and current guidelines are mainly based on expert consensus[2]. Patients are commonly prescribed inhaled, nebulised and oral medications and airway clearance physiotherapy, resulting in a **complex** and **time-consuming** treatment regimen which is usually prescribed twice daily and takes 30-45 minutes to complete each time. Furthermore, preparation of nebulised antibiotics (tobramycin and Colomycin®) is **complicated** and requires patients to be able to prepare the drugs prior to nebulisation and appropriately clean the equipment after use. There is, therefore, a high treatment burden associated with bronchiectasis.

There are no published studies specifically examining treatment adherence in bronchiectasis. However, research in other respiratory diseases such as cystic fibrosis[8], chronic obstructive pulmonary disease[9]. and asthma[10] consistently report sub-optimal adherence which negatively impacts on health outcomes[10–13]. There is no published data on predictors of adherence in bronchiectasis. However, data from other chronic diseases illustrate that it is a multi-faceted process which can be influenced by many different factors: patient-related, healthcare-related, socio-economic, therapy-related, condition-related[14].

Interventions are commonly used to influence behavioural change; such interventions are described as complex as they 'contain several interacting components'[15] and their development requires a theoretical and evidence-based approach involving all key stakeholders[15]. The Medical Research Council (MRC) has provided guidance on the

development and evaluation of complex interventions and states that there are four key elements: development, feasibility/piloting, evaluation and implementation[15]. We have outlined below the five stages of 'development' we have completed, based on this guidance.

APPROACH TO INTERVENTION DEVELOPMENT

Stage 1: Identifying a need

Aim: We determined rates of adherence to treatment, the relationship between adherence and health outcomes, and predictors of adherence behaviour.

Methods: We completed a prospective study of 75 patients with bronchiectasis in which we monitored patients' adherence to airway clearance and medications for bronchiectasis for one year.[16] We measured adherence using medication possession ratios (MPR) for inhaled antibiotics and other respiratory medicines. Self-reported adherence was used for airway clearance. We used an 80% cut-off to define adherence. We measured quality of life using the Quality of Life Questionnaire-Bronchiectasis (QOL-B)[17] and beliefs about treatment using the Beliefs about Medicines Questionnaire (BMQ).[18]

Key findings:

- 53% of patients were adherent to inhaled antibiotics and other respiratory medicines.[16]
- 41% of patients were adherent to airway clearance.[16]
- Only 16% were adherent to all prescribed treatments.[16]
- Non-adherent patients had significantly more pulmonary exacerbations.[16]
- Beliefs about treatment, age, treatment burden and quality of life predicted adherence to treatment.[16]

Conclusion: We established that there was a need to change adherence behaviour in bronchiectasis and had identified potentially modifiable factors that could be targeted in a future intervention.

Stage 2: Understanding what influences patients' adherence behaviour and identifying potential ways of changing this behaviour

Aim: We aimed to explore what influenced patients' adherence behaviour and identify potential components of an adherence intervention for bronchiectasis.

Methods: We completed semi-structured interviews with 16 patients with bronchiectasis. We transcribed interviews verbatim and analysed them using the Theoretical Domains Framework (TDF). The TDF is a framework of 12 domains, which was developed by experts in health psychology to link behaviour to psychological theories.[19] We coded all of the content of the interview transcripts to these domains and as a team, reached consensus on which domains were the most important. Following the identification of the important domains, three members of the team independently mapped the domains to a set of behaviour change techniques (BCTs).[20,21] BCTs are 'an observable, replicable, and irreducible component of an intervention designed to alter or redirect causal processes that regulate behavior; that is, a technique is proposed to be an "active ingredient" (e.g., feedback, self-monitoring, and reinforcement). BCTs can be used alone or in combination and in a variety of formats.'

Key findings:

- 8 domains were identified as influencing patient adherence behaviour: knowledge, skills, beliefs about capabilities, beliefs about consequences, motivation, social influences, behavioural regulation and nature of behaviour.
- 23 BCTs were identified that could potentially change patient adherence behaviour.

Conclusion: We have identified what influences patients' adherence behaviour and have identified a list of components that could be used as part of an adherence intervention in this population.

Stage 3: Understanding healthcare professionals’ perspectives on patient adherence behaviour

Aim: We aimed to identify healthcare professionals’ views on adherence in bronchiectasis including methods for improving adherence in this population and healthcare professionals’ challenges in managing adherence in bronchiectasis.

Methods: We completed focus groups and interviews with 46 primary and secondary care healthcare professionals from across Northern Ireland. We transcribed interviews verbatim and analysed them using the TDF and identified BCTs as described in Stage 2.

Key findings:

- 8 domains were identified as influencing healthcare professionals’ ability to manage patient adherence behaviour: knowledge, skills, beliefs about capabilities, beliefs about consequences, motivation, social influences, behavioural regulation and nature of behaviour.
- 26 BCTs were identified that could potentially change healthcare professional's ability to manage adherence.

Conclusion: We have identified what affects healthcare professionals’ ability to manage patients’ adherence, which could be used to develop a training package for healthcare professionals to facilitate them to deliver a behaviour change intervention for patients.

Stage 4: Identifying the existing evidence base for adherence interventions in chronic respiratory disease

Aim: To identify components of effective adherence interventions in chronic respiratory disease.

Methods: We completed a systematic review. We searched the Cochrane Central Register of Controlled Trials (CENTRAL), MEDLINE, EMBASE, CINAHL, International Pharmaceutical Abstracts, PsycINFO, Sociological abstracts and PEDro (using disease-specific and adherence search terms) from their inception until February 2013. Randomised controlled trials comparing any adherence intervention to another adherence intervention, no intervention

or usual care in adults (18 years or older), with a clinical diagnosis of chronic respiratory disease based on the Cochrane Airways Group Trials register (asthma, bronchiectasis, chronic obstructive pulmonary disease [COPD], allergic bronchopulmonary aspergillosis [ABPA], interstitial lung disease, sleep apnea) plus cystic fibrosis were included. Two review authors independently reviewed returned articles, extracted data and assessed risk of bias using the Cochrane Risk of Bias tool. We completed a narrative synthesis of included studies.

Key findings:

- 51 studies were included in the final review and most studies had a high risk of bias.
- 16 studies improved adherence (measured by objective measures) and 14 studies did not improve adherence (measured by objective measures).
- 8 (50%) studies deemed to be effective interventions that were developed using psychological theory. Only 3 studies (21%) which deemed interventions ineffective used psychological theory in their development.
- Effective interventions contained multiple components and generally included an information component alongside multiple other practical components such as goal setting, problem-solving, feedback and action planning.
- Ineffective interventions tended to have fewer components than those deemed effective and often focused on information transfer without practical strategies to alter adherence behaviour.
- Effective interventions were more tailored to the individual.
- There was no clear evidence for the most effective way to deliver these interventions in terms of format, mode of delivery or healthcare context.

Conclusion: Interventions which are underpinned by psychological theory, use multiple practical components that go beyond education and can be tailored to the individual appear to be the most effective in changing adherence behaviour in chronic respiratory disease.

Stage 5: Triangulation of the evidence from Stage 1-4

Aim: We sought to bring together all of the data from the previous four stages to develop a final list of BCTs and bronchiectasis-specific examples for these BCTs to form a draft patient adherence intervention.

Method: We reviewed the BCTs indentified in Stage 2 and 3. We ranked these BCTs using a published scoring system.[20] We reached consensus within the team about which BCTs should be included in the final patient list and final healthcare professional list based on these scores and data collected in the systematic review. From these two lists, we developed a single list of BCTs which were common across the two groups. These lists of BCTs did not contain specific content for patients with bronchiectasis and their healthcare professionals. We generated bronchiectasis-specific content using data collected during Stage 2 and 3.[21]

Key findings:

- 12 common BCTs were identified e.g. 'Persuasive communication'
- 12 bronchiectasis-specific examples were generated e.g. 'Expert patient or lead consultant endorses the importance of adherence to airway clearance'

Conclusion: We have developed a draft bronchiectasis-specific adherence intervention containing 12 BCTs. Input is now needed from experts to determine how this intervention can be delivered to patients by healthcare professionals.

RESEARCH OUTPUTS

Full publications for these studies are in preparation. Preliminary findings have been presented at numerous local, national and international conferences:

Conference	Presentation	Location and year
American Thoracic Society	Poster discussion	San Diego, May 2014
Irish Thoracic Society	Invited speaker	Londonderry, November 2013
Ulster Society of Internal Medicine	Oral	Belfast, October 2013
Royal Pharmaceutical Society[22]	Poster	Birmingham, Sept 2013
Rehabilitation and Therapy Research Society[23,24]	Oral	University of Ulster, June 2013
American Thoracic Society[16,25]	Oral	Philadelphia, May 2013
Cochrane in Ireland	Poster	Belfast, January 2013
British Thoracic Society[26]	Poster	London, December 2012
British Thoracic Society[27]	Poster	London, December 2011
Irish Society of Chartered Physiotherapists	Poster	Mullingar, November 2011
All Ireland Pharmacy Conference	Poster	Dublin, April 2011

REFERENCES

1. Barker AF. Bronchiectasis. N Engl J Med 2002;346(18):1383–1393.

2. Pasteur MC, Bilton D, Hill AT. Guideline for non-CF bronchiectasis. Thorax 2010;65:Suppl 1.

3. Keistinen T, Säynäjäkangas O, Tuuponen T, Kivelä S-L. Bronchiectasis: an orphan disease with a poorly-understood prognosis. Eur Respir J 1997;10(12):2784–2787.

4. Martínez-García MA, Soriano JB. Physiotherapy in bronchiectasis: we have more patients, we need more evidence. Eur Respir J 2009;34(5):1011–2.

5. O'Donnell AE. Bronchiectasis. Chest 2008;134(4):815–823.

6. Weycker D, Edelsberg J, Oster G, Tino G. Prevalence and economic burden of bronchiectasis. Clin Pulm Med 2005;12(4):205–209.

7. Roberts HJ, Hubbard R. Trends in bronchiectasis mortality in England and Wales. Respir Med 2010;104:981–985.

8. Myers LB, Horn SA. Adherence to chest physiotherapy in adults with cystic fibrosis. J Health Psychol 2006;11:915–926.

9. Latchford G, Duff A, Quinn J, Conway S, Conner M. Adherence to nebulised antiobiotics in cystic fibrosis. Patient Educ Couns 2009;75:141–144.

10. Gamble J, Stevenson M, McClean E, Heaney L. The prevalence of nonadherence in difficult asthma. Am J Respir Crit Care Med 2009;180:817–822.

11. Eakin MN, Bilderback A, Boyle MP, Mogayzel PJ, Riekert KA. Longitudinal association between medication adherence and lung health in people with cystic fibrosis. J Cyst Fibros 2011;10(4):258–64.

12. Quittner AL, Zhang J, Marynchenko M, et al. Pulmonary medication adherence and healthcare utilization in cystic fibrosis. Chest 2014;doi:10.1378/chest.13-1926[online first].

13. Vestbo J, Anderson JA, Calverley PMA, et al. Adherence to inhaled therapy, mortality and hospital admission in COPD. Thorax 2009;64(11):939–943.

14. World Health Organisation. Adherence to long-term therapies: evidence for action [Internet]. 2003. Available from: http://www.who.int/chp/knowledge/publications/adherence_report/en/

15. Medical Research Council. Developing and evaluating complex interventions: new guidance [Internet]. 2008. Available from: http://www.mrc.ac.uk/Utilities/Documentrecord/index.htm?d=MRC004871

16. McCullough AR, Hughes C, Tunney MM, Elborn JS, Quittner AL, Bradley JM. Treatment adherence and health outcomes in patients with bronchiectasis infected with Pseudomonas aeruginosa. Am J Respir Crit Care Med 2013;187:A5231.

17. Quittner AL, Marciel KK, Salathe MA, et al. A preliminary Quality of Life Questionnaire-Bronchiectasis: a patient-reported outcome measure for bronchiectasis. Chest 2014; doi:10.1378/chest.13-1891[online first].

18. Horne R, Weinman J, Hankins M. The beliefs about medicines questionnaire: The development and evaluation of a new method for assessing the cognitive representation of medication. Psychol Health 1999;14(1):1–24.

19. Michie S, Johnston M, Abraham C, et al. Making psychological theory useful for implementing evidence based practice: a consensus approach. Qual Saf Health Care 2005;14(1):26–33.

20. Michie S, Johnston M, Francis J, Hardeman W, Eccles M. From Theory to Intervention: Mapping Theoretically Derived Behavioural Determinants to Behaviour Change Techniques. Appl Psychol 2008;57(4):660–680.

21. Michie S, Richardson M, Johnston M, et al. Behavior Change Technique Taxonomy. Ann Behav Med 2013;46(1):81–95.

22. McCullough A, Ryan C, Bradley J, O’Neill B, Elborn S, Hughes CM. “If you don't know about it, it’s not a problem": healthcare professionals’ views on barriers to treatment adherence and strategies to improve adherence in adults with bronchiectasis. Int J Pharm Pract 2013;21(Suppl 2):38.

23. McCullough A, Hughes C, Tunney M, Elborn J, Quittner A, Bradley J. Treatment adherence and health outcomes in patients with bronchiectasis infected with Pseudomonas aeruginosa. Phys Ther Rev 2014;19(1).

24. McCullough AR, Tunney MM, Elborn JS, Bradley J, Hughes C. Patients’ perspectives on decision-making in adherence to treatment in bronchiectasis. Phys Ther Rev 2014;19(1).

25. McCullough A, Tunney M, Elborn J, Bradley J, Hughes C. Patients’ perspectives on decision-making in adherence to treatment in bronchiectasis. Am J Respir Crit Care Med 2013;187:A5229.

26. McCullough A, Tunney M, Hughes CM, Elborn J, Bradley J. Adherence to treatment in patients with bronchiectasis infected with Pseudomonas aeruginosa. Thorax 2012;67(Suppl 2):A142.

27. McCullough A, Tunney M, Hughes CM, Elborn J, Bradley J. Assessment of health-related quality of life in non-cystic fibrosis bronchiectasis using a new disease-specific tool for measuring HRQOL: the Quality of Life- Bronchiectasis (QOL-B) questionnaire. Thorax 2011;66(Suppl 4):P249.
