# Supplementary material for: Defining the content and delivery of an intervention to Change AdhereNce to treatment in BonchiEctasis (CAN-BE): a qualitative approach incorporating the Theoretical Domains Framework, behavioural change techniques and stakeholder expert panels
Source: BMC Health Serv Res. 2015 Aug 22;15:342. doi: 10.1186/s12913-015-1004-z (PMC4546345; doi:10.1186/s12913-015-1004-z)
Supplement: Additional file 5: — Materials used during patient expert panel. (PDF 1477 kb) [file 12913_2015_1004_MOESM5_ESM.pdf]

Queen's University Belfast and Ulster University logos here

Development of a bronchiectasis-specific adherence intervention:

‘All medicine, all illness is personal to that individual’

Dr Amanda McCullough, Dr Cristín Ryan, Dr Brenda O’Neill, Prof. Stuart Elborn, Prof. Judy Bradley and Prof. Carmel Hughes

April 28th 2014

Agenda

9.30-10.00am	Refreshments and consent
10.00-10.20am	Welcome and introduction
10.20-10.50am	Presentation: *How did we create our plan to make it easier for patients to do their treatments?*
10.50-11.00pm	Task 1: *Does this plan look like something that would be helpful?*
11.00-11.15am	Coffee
11.15-12.15pm	Task 2: Group discussion: *How can we use this plan in everyday life?*
12.15-12.45pm	Feedback session on discussion
12.45-1.00pm	Close

Team

- Team of three physiotherapists, two pharmacists and one doctor
- Based at Queen's University, University of Ulster and Belfast City Hospital
- Experienced in treating patients with bronchiectasis
- Experienced in doing research with patients with bronchiectasis
- Work closely with healthcare professionals from across Northern Ireland
- Involved in the development of services for bronchiectasis across Northern Ireland

Queens University Belfast and Ulster University logos here

Development of a bronchiectasis-specific adherence intervention:

Session 1: How did we create our plan to make it easier for patients to do their treatments?

Dr Amanda McCullough

April 28th 2014

Plan for this talk

- What is the goal of today's meeting?
- Definitions
- Why did we create this plan for bronchiectasis?
- How did we create this plan?
- What does this plan look like?

What is the goal of today's meeting?

We want to find out:

1. If you think this plan looks like it would be helpful

2. How we could use this plan in everyday life

Definitions

Adherence	Doing your treatments for bronchiectasis as prescribed by your healthcare professional.
Behaviour	The way we do things.
Behaviour change techniques (BCTs)	Things that a person can do to change the way they do things.
Bronchiectasis	Damage to the airways that can lead to your lungs making too much mucus. Mucus can build up and lead to chest infections.
Intervention	A list of behaviour change techniques that can be used together to change a person’s behaviour.

Why did we create this plan for bronchiectasis?

- Less than 50% of patients do treatments that are prescribed
- ‘Drugs don't work in patients who don't take them’ - C. Everett Koop, M.D.
- Missing treatments can lead to more chest infections, more time in hospital or worse quality of life

Why did we create this plan for bronchiectasis?

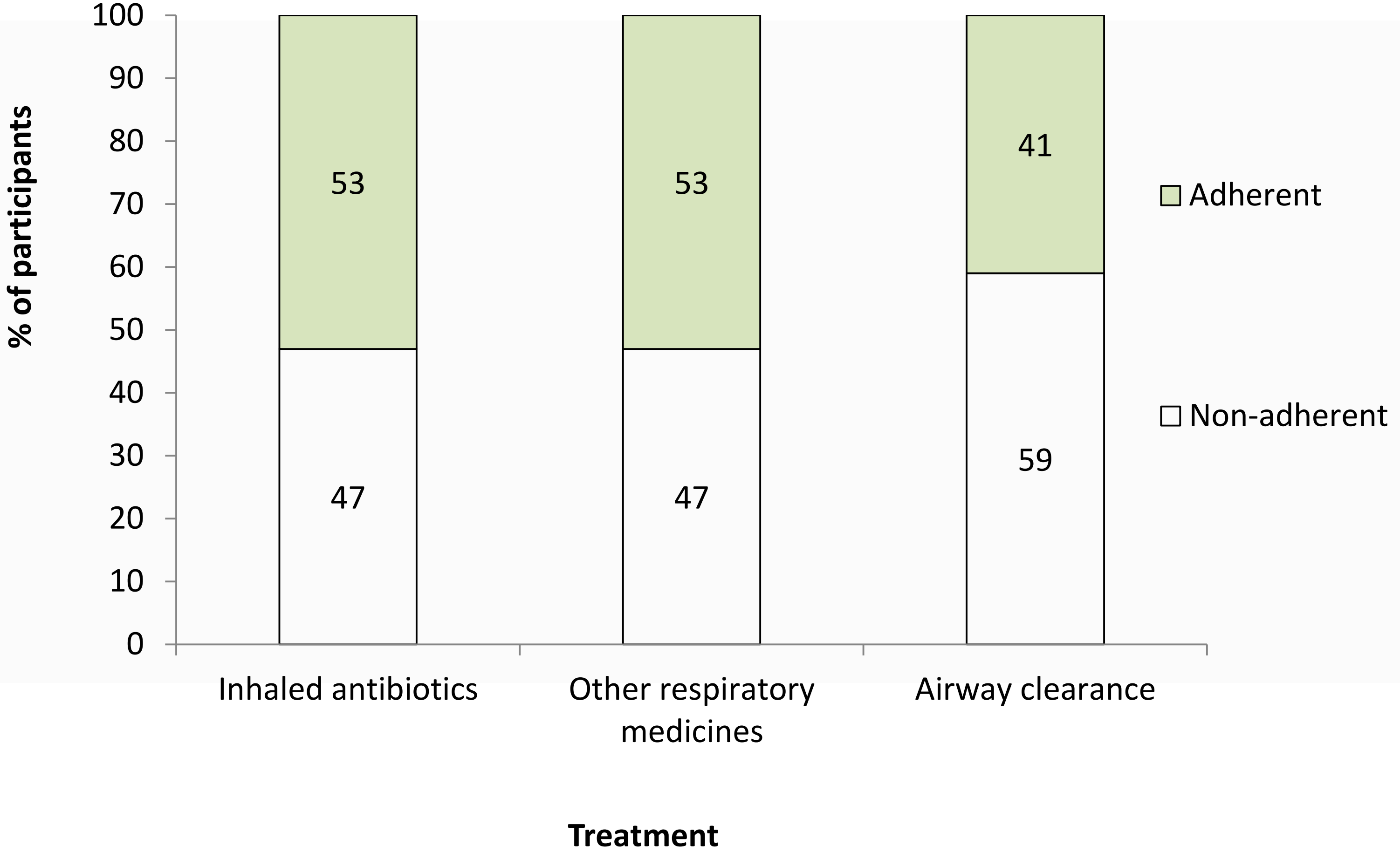

How did we create this plan?

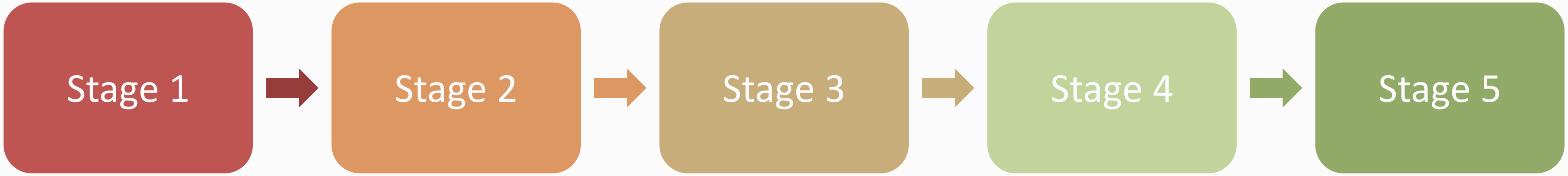

Stage 1: Understanding whether a patient is likely to do a treatment

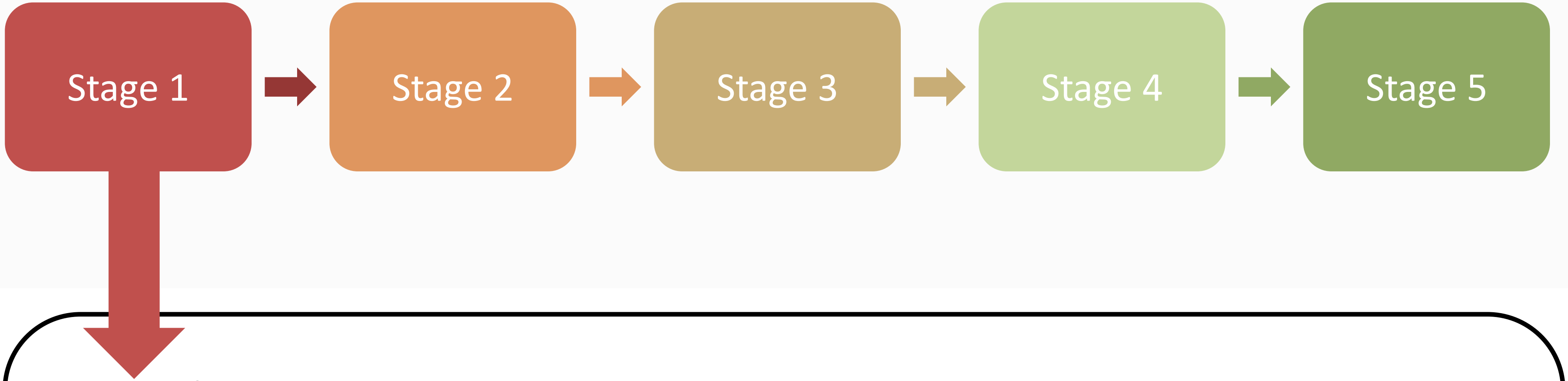

Key results:

- Patients who believed they needed airway clearance were more likely to do this treatment.
- Patients who were concerned about side effects of medications were less likely to take them
- Older patients were more likely to take medications and do airway clearance
- Patients who were prescribed more medications were less likely to take them

Stage 2: Deeper understanding of reasons for not doing treatment

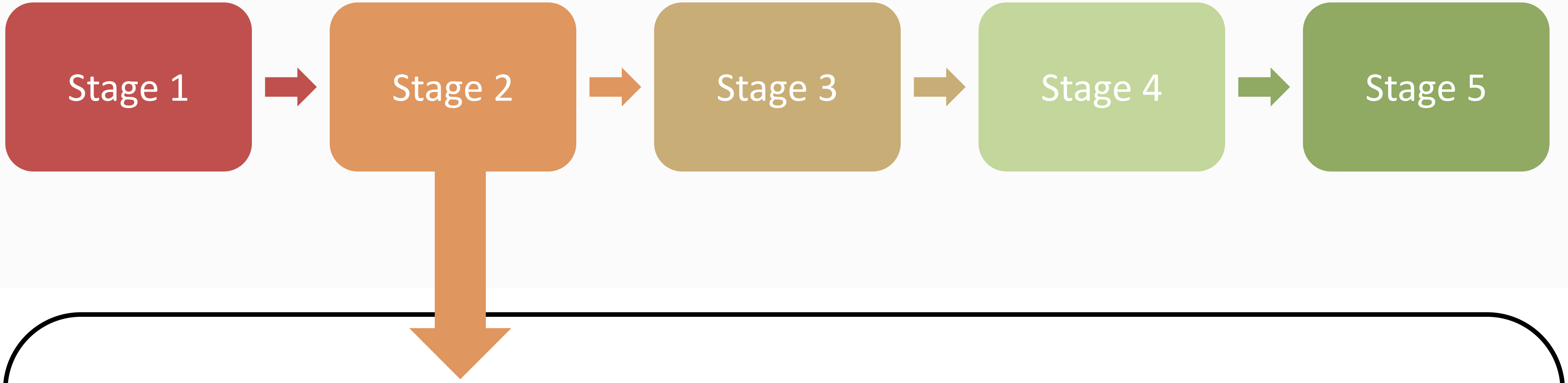

Key results:

- Patient-specific barriers and motivators
- 8 important things that influenced whether they did a treatment
- 23 ways to change behaviour found

Stage 3: Healthcare professionals' views on what could make it easier for patients

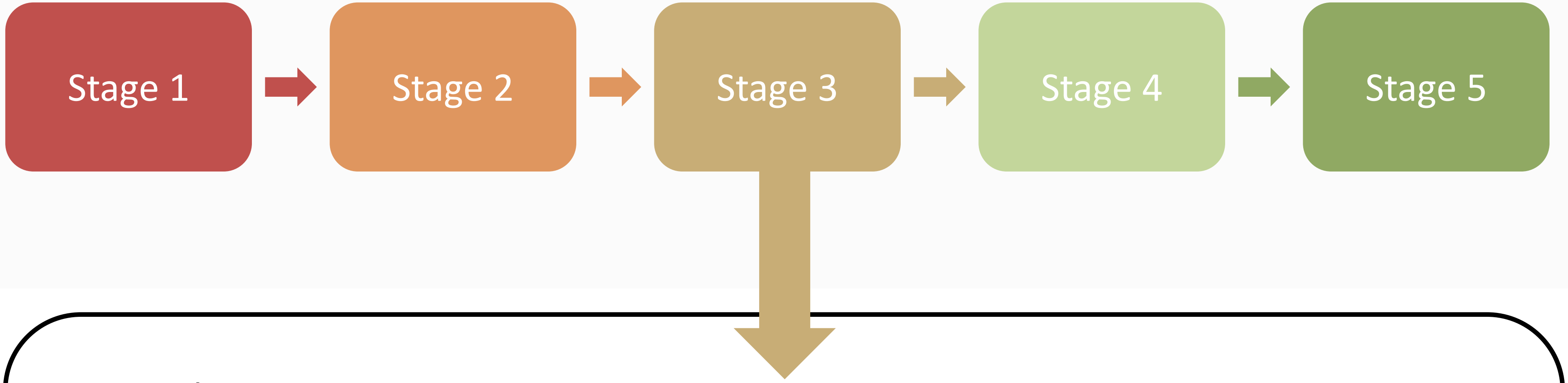

Key results:

- Patients need more education on bronchiectasis and its treatment
- Patients need information about the negative consequences of not doing treatments
- Using self-management plans helps patients to take their treatments
- Better communication is needed between patients and healthcare professionals.

Stage 4: Finding out what other research had been done

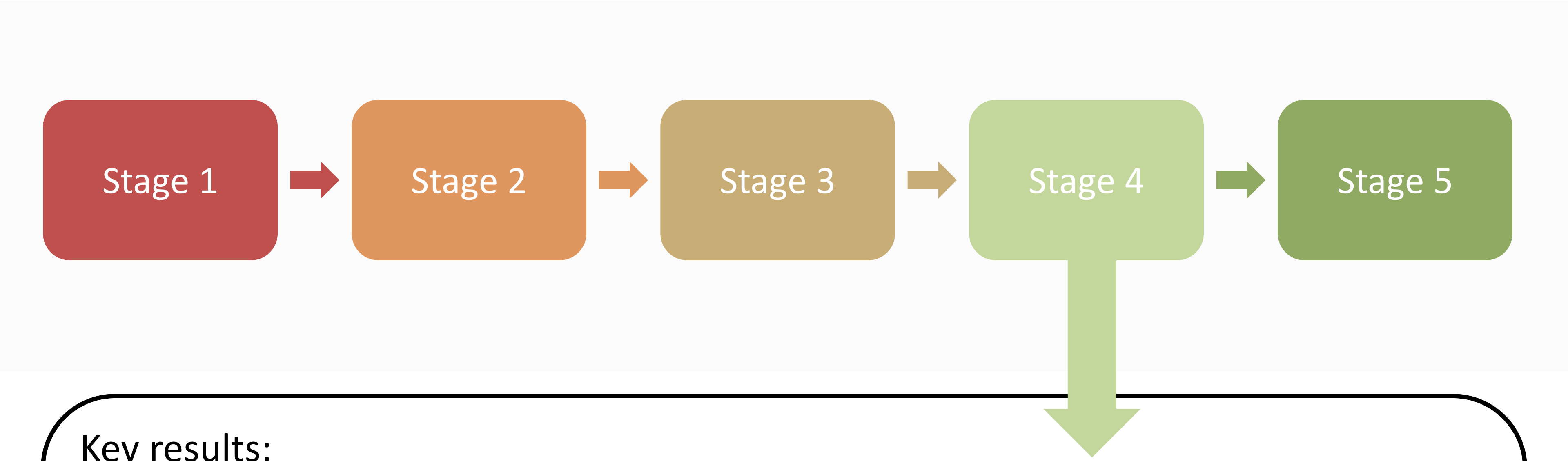

Key results:

- 51 research studies
- Studies that were effective used a number of ways to change patients' behaviour
- Most studies educated patients about their disease and treatment
- However, they also used practical ways of changing behaviour such as, setting goals with patients, planning how to deal with changes in treatment and solving problems that patients faced

Stage 5: Bringing together information from Stages 1 to 4

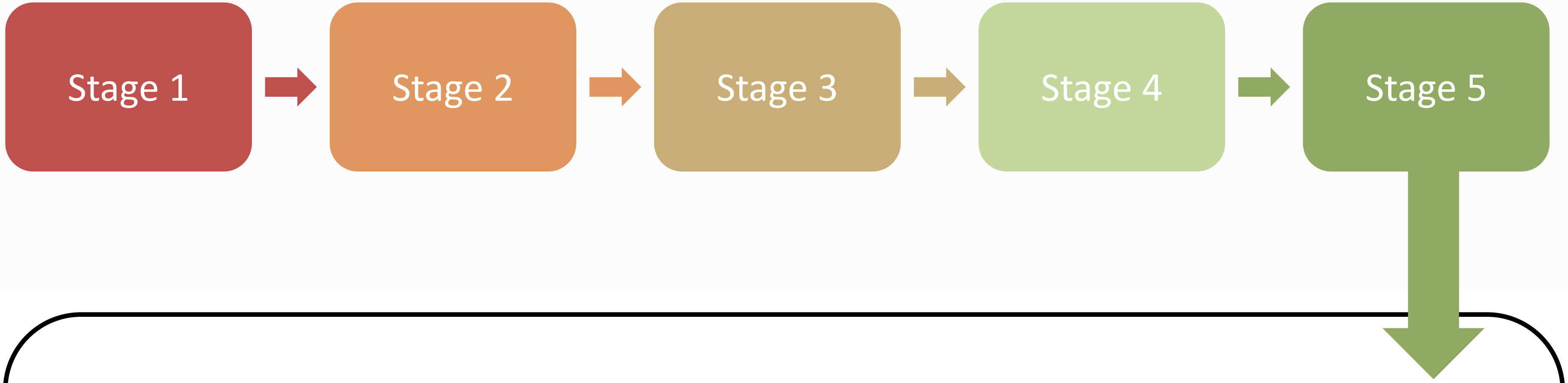

Key results:

- Plan to change behaviour includes 12 ways to change behaviour (BCTs)
- Need to get patients' views on how we could use this in everyday life

Content of the plan

Monitoring

Self-monitoring

Feedback

Information about behaviour

Social support

- Pick and choose which ones you want to use
- Tailored to patient
- Tailored to treatment

Persuasive communication

Role play

Cognitive restructuring

Graded task

Action planning

Goal specified

Problem-solving

Some examples…

Behavioural change technique	• Self-monitoring
Description	• Record your own behaviour
What you might do	• Record your symptoms, how often you take a treatment and how long you spend on a treatment at the end of each day for a certain period of time e.g. one week

Some examples…

Behavioural change technique	• Persuasive communication
Description	• A respected person tells you the reasons to change your behaviour
What you might do	• Your consultant or another patient tells you about why you should do your treatments (medication and airway clearance) as prescribed

Real life example…

"Sometimes, I just can't be bothered (taking inhaled antibiotics) *and I know I should…I do feel better, overall, when I take it and I know I need to take it. But I just, sometimes, just can't be bothered at night time going through the whole rigmarole." (F11NA)*

In this example, we might have used:

- Self-monitoring
- Problem-solving
- Goal setting
- Action planning

Real life example…

"If I don't feel I've got it (mucus), *I don't use the Acapella®." (M5NA)*

In this example, we might have used:

- Persuasive communication
- Information about behaviour/outcome
- Self-monitoring

Summary

- Patients who do not take treatments have more chest infections
- Plan needed to make it easier for patients to do treatments
- Plan developed with patients and health professionals, and using existing research
- Plan includes:
  - 12 behavioural change techniques
  - Tailored to individual patients
  - Tailored to treatments

'All medicine, all illness is personal to that individual'

- We know **what** will be included, now we need to know **how** to do it

Does this plan look like something that would be helpful?

Tea break

Queen's University and Ulster University logos here

Development of a bronchiectasis-specific adherence intervention:

Session 2: Group discussion - How can we use this plan in everyday life?

Dr Amanda McCullough

April 28th 2014

Outline of group discussion

- You should each have:
  - A list of questions
  - A list of definitions
  - A table showing each of the 12 parts of the plan
  - A page describing what we already know about how we might be able to deliver this plan in everyday life
- Read information
- Talk to each other about the questions
- Please ask if you have any questions

Queen's University Belfast and Ulster University logos here

Development of a bronchiectasis-specific adherence intervention:

Session 3: Feedback group discussion

28th April 2014

Feedback on small group task

- Which healthcare professionals should deliver this intervention (plan)?
- How often should you get this intervention?
- How long should you get this intervention for?
- What format should this intervention take?
- Where should this intervention take place?
- How would you know if you the intervention was working?

Queen's University Belfast and Ulster University logos here

Development of a bronchiectasis-specific adherence intervention:

Session 4: Close

Dr Amanda McCullough

April 28th 2014

Future plans

- Summarise feedback and send to you
- Know what the intervention is going to include and how we could use it in everyday life
- Develop things to use to deliver the intervention e.g. training for healthcare professionals
- Test in a small research study
- Test in a large research study across the UK

Questions?

Thank you

Funders

Funder logos here

Acknowledgements

Dr Michael Tunney, Prof. Alexandra Quittner, Dr Ian Bradbury and all of the staff and patients who participated in this research

Bronchiectasis meeting

Monday 28th April 2014

Room N09/10, School of Pharmacy, Queen's University Belfast

Programme for meeting

9.30-10.00am	Refreshments and consent
10.00-10.20am	Welcome and introduction
10.20-10.50am	Presentation: *How did we create our plan to make it easier for patients to do their treatments?*
10.50-11.00pm	Task 1: *Does this plan look like something that would be helpful?*
11.00-11.15am	Coffee
11.15-12.15pm	Task 2: Group discussion: *How can we use this plan in everyday life?*
12.15-12.45pm	Feedback session on discussion
12.45-1.00pm	Close

Group task

Please read the information we have given you about the plan (intervention). Look at the questions below and talk about them with the rest of the group. We will give you time to talk to each other. After 15 to 20 minutes, we will come over to see how you are getting on. If you any questions, please just ask. There are no right or wrong answers and we are interested in everyone's opinion. We will write down some short notes about what the group are saying for us all to talk about more later on.

1. Which patients should get this intervention?
   e.g. all patients, those who are sicker, those who have difficulty doing their treatments

2. Which healthcare professionals should deliver this intervention?
   e.g. physiotherapist, nurse, doctor, pharmacist, psychologist, other

3. How often should you get this intervention?
   e.g. every time you see your healthcare professional, every year

4. How long should you get this intervention for?
   e.g. only once or more than once

5. What format should this intervention take?
   e.g. written information, spoken information, using the internet, in a group with other patients, one-to-one with a healthcare professional

6. Where should this intervention take place?
   e.g. at home, in hospital, in GP surgery, pharmacy

7. How would you know if the intervention was working?
   e.g. able to take more medication, have fewer chest infections

Definitions

Adherence	Doing your treatments for bronchiectasis as prescribed by your healthcare professional.
Behaviour	The way we do things.
Behaviour change techniques (BCTs)	Things that a person can do to change the way they do things.
Bronchiectasis	Damage to the airways that can lead to your lungs making too much mucus. Mucus can build up and lead to chest infections.
Intervention	A list of behaviour change techniques that can be used together to change a person's behaviour.

Intervention to make it easier for patients to take treatments for bronchiectasis

We have used our research to design a plan (intervention) for patients with bronchiectasis. The intervention has up to 12 different parts, known as behaviour change techniques. Usually only a few of these will be used at one time. This intervention can be tailored to you. Your healthcare professional will only choose a couple of things from it that they think will help you specifically.

Please read the table below. The first column tells you about each of the 12 behaviour change techniques that could be used. The second column describes what you or your health professional might do if you used this part of the intervention. The information in the second column is just an example of what you or your healthcare professional might do. These examples can be changed to suit your needs.

Behaviour change technique	What you or your healthcare professional would do
**Monitoring:** with your permission, someone records your behaviour	Your healthcare professional will collect information on how often you take a specific treatment and how this affects your health e.g. the number of chest infections you have during a certain time period.
**Self-monitoring:** record your own behaviour	Depending on treatment e.g. airway clearance or medication. Record your symptoms, how often you take a treatment and how long you spend on a treatment at the end of each day for a certain period of time e.g. one week.
**Feedback:** you are given information about your behaviour	Your healthcare professional tells you about how often you take a specific treatment and how this has affected your health during a certain period of time e.g. past year.
**Information about behaviour:** you are given information about your behaviour and the consequences of that behaviour	Your healthcare professional will explain to you the link between missing treatments and how this affects your health e.g. going into hospital more, having to have more antibiotics and other treatments or having worse symptoms.
**Persuasive communication:** a respected person tells you the reasons to change your behaviour	Your consultant or another patient tells you about why you should do your treatments (medication and airway clearance) as prescribed.

Behaviour change technique	What you or your healthcare professional would do
**Cognitive restructuring:** change the way you think about the reasons for your behaviour and the consequences of it	Think about the behaviour you want to change (e.g. missing medication or airway clearance). Rather than thinking of ways of increasing the numbers of days you do a treatment (medication/airway clearance), think of it as reducing the number of days you miss a treatment.
**Problem solving:** explore reasons for your behaviour and look at ways you can overcome these problems.	Identify specific reasons for finding it difficult do treatments as prescribed and think of ways to overcome these problems. Think of what you could do if you were unable to do your treatments for some reason.
**Goal setting:** agree a goal for the behaviour you would like to change	Agree a daily goal (e.g. do airway clearance once per day) with your healthcare professional.
**Action planning:** make a detailed plan of how you will perform the behaviour you would like to change	Create a plan with your healthcare professional for achieving the goal you have set. Plan how you will achieve this goal on certain days of week, when you will fit in your treatment and where you will do your treatment.
**Graded tasks:** start with easy tasks and gradually make them more difficult until you change your behaviour	Think about the behaviour you want to change (e.g. missing medication or airway clearance) and how much you do it at present. Take your medications/do airway clearance one day per week in the first week; 2 days per week in the second week and so on until you reach your goal.
**Role-play:** practise how you would behave in a pretend situation	Show your healthcare professional how you take your medication/perform airway clearance. Your healthcare professional will show you the correct way to do these treatments and will watch you performing this for them
**Social support:** receive encouragement from others (e.g. from friends, family, healthcare professionals other patients) to change your behaviour	Arrange for a family member (e.g. husband or wife) to encourage you to stick with the behaviour change programme.

What do we already know about how this intervention could be used in everyday life?

Patients thought that the intervention could:

- Be led by a knowledgeable healthcare professional.
- Be reinforced by different healthcare professionals.
- Include reviews every 3 months that could be increased or decreased depending on what patients needed.
- Be either one-to-one with their healthcare professional or in a group.
- Include information that was written, verbal or shown on posters or diagrams.
- Include information which uses everyday language that patients could understand

Healthcare professionals thought that the intervention could:

- Be tailored to individual patients' needs at different times during their disease.
- Prioritise sicker patients or those who were struggling to do treatments.
- Be reinforced by different healthcare professionals.
- Include healthcare professionals working as a team including GPs and hospital healthcare professionals.
- Be either one-to-one with your healthcare professional or in a group. They thought groups may be better in a hospital setting.
- Include information that was written, verbal or shown on posters or diagrams.
- Include information which uses everyday language that patients could understand
