# Supplementary material for: Defining the content and delivery of an intervention to Change AdhereNce to treatment in BonchiEctasis (CAN-BE): a qualitative approach incorporating the Theoretical Domains Framework, behavioural change techniques and stakeholder expert panels
Source: BMC Health Serv Res. 2015 Aug 22;15:342. doi: 10.1186/s12913-015-1004-z (PMC4546345; doi:10.1186/s12913-015-1004-z)
Supplement: Additional file 6: — Materials used in HCP expert panel. (PDF 1387 kb) [file 12913_2015_1004_MOESM6_ESM.pdf]

Queen's University Belfast and Ulster University logos

Development of a bronchiectasis-specific adherence intervention:

'All medicine, all illness is personal to that individual'

Dr Amanda McCullough, Dr Cristín Ryan, Dr Brenda O'Neill, Prof. Stuart Elborn, Prof. Judy Bradley and Prof. Carmel Hughes

May 1st 2014

Team

- Multidisciplinary team with academic and clinical expertise
- Expertise in the development of complex interventions using psychological theory
- Long-standing clinical links with the Regional Bronchiectasis Centre (managing >1000 patients with bronchiectasis)
- Co-Leads of Respiratory and Primary Care Clinical Research Networks
- Extensively involved in the development of Standards of Care for bronchiectasis in Northern Ireland

Agenda

9.30-10.00am	**Refreshments and consent**
10.00-10.30am	Welcome and introduction
10.30-11.15am	Background to adherence intervention development and outline of patient adherence intervention
11.15-11.30am	**Coffee**
11.30-1.00pm	Small group session: *Defining how the patient adherence intervention could be delivered and how healthcare professionals could be trained to deliver it*
1.00pm-1.45pm	**Lunch**
1.45pm-3.30pm	Feedback and discussion from the small group session
3.30-4.00pm	**Close**

Queen's University Belfast and Ulster University logos

Development of a bronchiectasis-specific adherence intervention:

Session 1: Background to adherence intervention development

Dr Amanda McCullough

May 1st 2014

Outline

- Aims and outcomes
- Rationale for intervention development
- Approach to intervention development
- Outline of proposed patient intervention
- Small group task
- Feedback to the group

Aim and outcomes

- We aim to obtain your views on:
  - Our approach to the intervention development
  - How this proposed intervention could be delivered
  - How healthcare professionals could be trained to deliver this intervention

- By the end of the session we should have:
  - Reached agreement on how this intervention could be delivered
  - Reached agreement about how healthcare professionals could be trained to deliver this intervention

Rationale – why bronchiectasis?

Logos of relevant research papers

Rationale – why adherence?

‘Drugs don't work in patients who don't take them’
- C. Everett Koop, M.D.

Ventolin inhaler	1542%
Azithromycin	74%
Mucodyne	71%
Tobramycin nebuliser	24%
Symbicort inhaler	8%
Airway clearance	never

Rationale – why adherence?

"Sometimes, I just can't be bothered (taking inhaled antibiotics) *and I know I should…I do feel better, overall, when I take it and I know I need to take it. But I just, sometimes, just can't be bothered at night time going through the whole rigmarole." (F11NA)*

Approach to intervention development

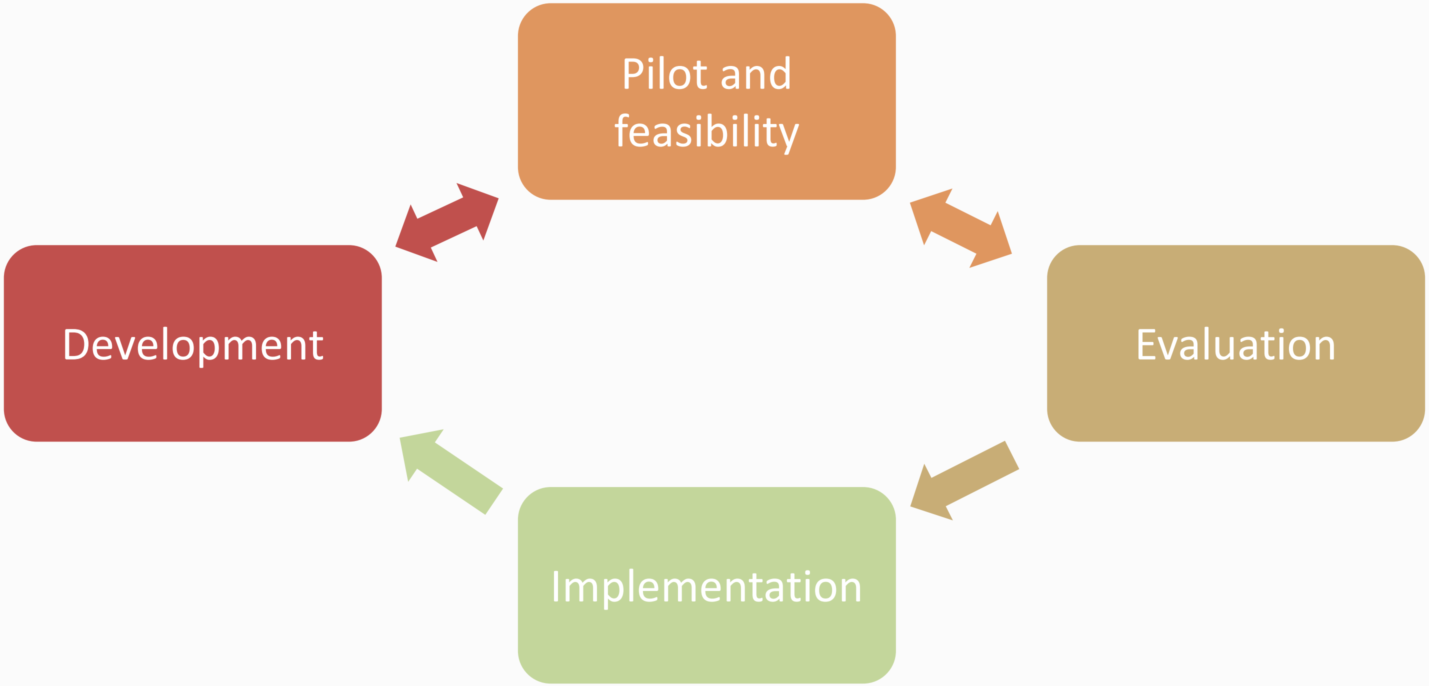

Theoretical domains framework (TDF)

12 domains:

- Knowledge
- Skills
- Social/professional role/identity
- Beliefs about capability
- Beliefs about consequences
- Motivation
- Memory, attention, decision-making processes
- Environmental context
- Social influences
- Emotion
- Behavioural regulation
- Nature of behaviour

Screen shot of:
Michie et al. Making psychological theory useful for implementing evidence based practice: a consensus approach. Qual Saf Health Care 2005; 14: 26-33

Behavioural change techniques (BCTs)

‘An observable, replicable, and irreducible component of an intervention designed to alter or redirect causal processes that regulate behavior; that is, a technique is proposed to be an “active ingredient” (e.g., feedback, self-monitoring, and reinforcement).’

Screenshot of:

Michie et al. From thoery to intervention: mapping theoretically derived behavioural determinants to behaviour change techniques. Applied Psychology 2008; 37 (4): 660-680

Michie et al. The behavior change technque taxonomy (v1) of 93 heirarchically clustered techniques: building an international consensus for the reporting of behaviour change interventions. Annals of Behav Med 2013; 46 (1): 81-95

Mapping TDF to BCTs

	TDF							
**BCT**	Knowledge	Skills	Capability	Consequences	Motivation	Social	Behavioural regulation	Nature of behaviour
Goal/target specified: behaviour or outcome	000	020	322	020	323	000	131	011
Monitoring	011	023	022	121	321	000	131	021
Self-monitoring	011	033	022	221	321	000	131	021

Development phase

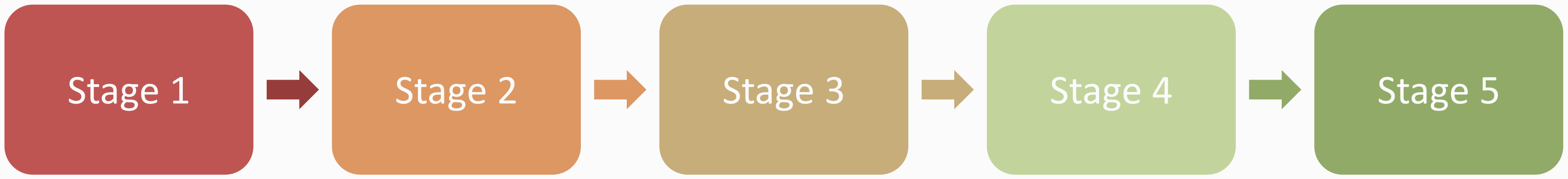

Stage 1: Identifying a need

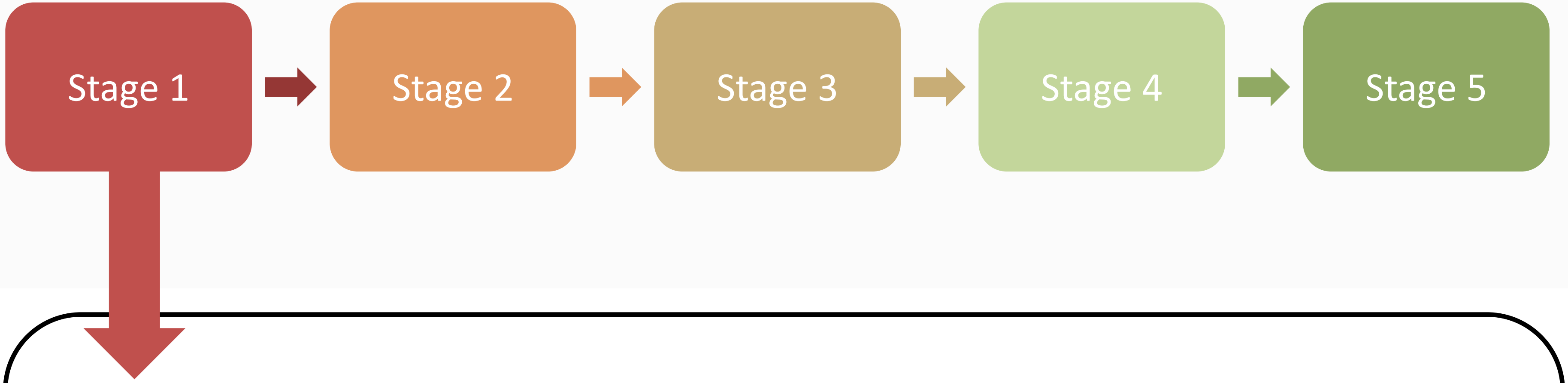

Key findings:

- ~ 50% adherence
- Adherence lowest for airway clearance
- Non-adherent = more pulmonary exacerbations
- Beliefs about treatment, age, treatment burden and quality of life predicted adherence

Stage 2: Understanding what influences patient behaviour

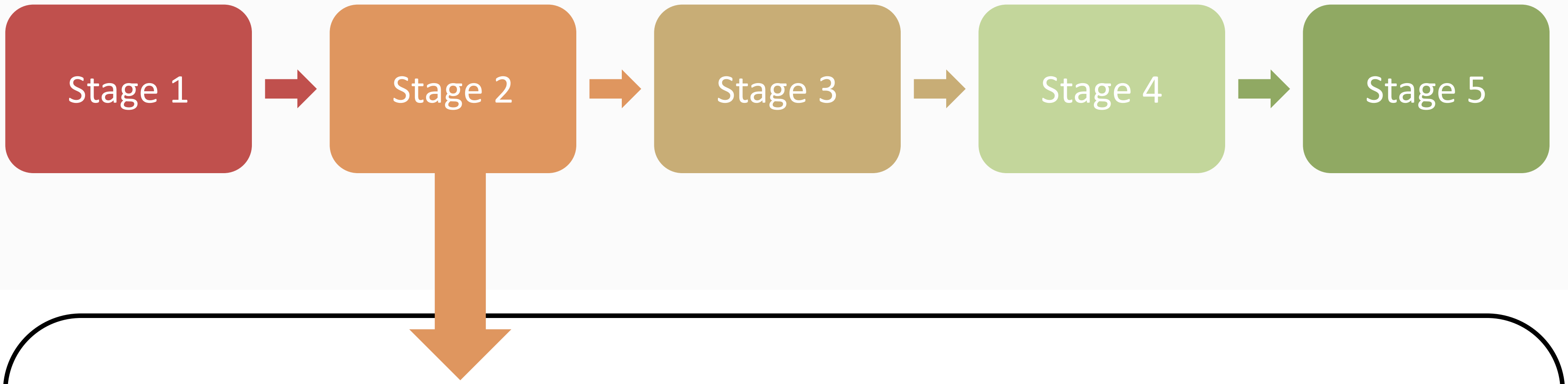

Key findings:

- Patient-specific combination of barriers and motivators
- 8 important TDF domains: Knowledge, Skills, Beliefs about capability, Beliefs about consequences, Motivation, Social, Behavioural regulation, Nature of behaviour
- 23 BCTs identified that could potentially change patient adherence behaviour

Stage 3: Understanding healthcare professionals' views on adherence

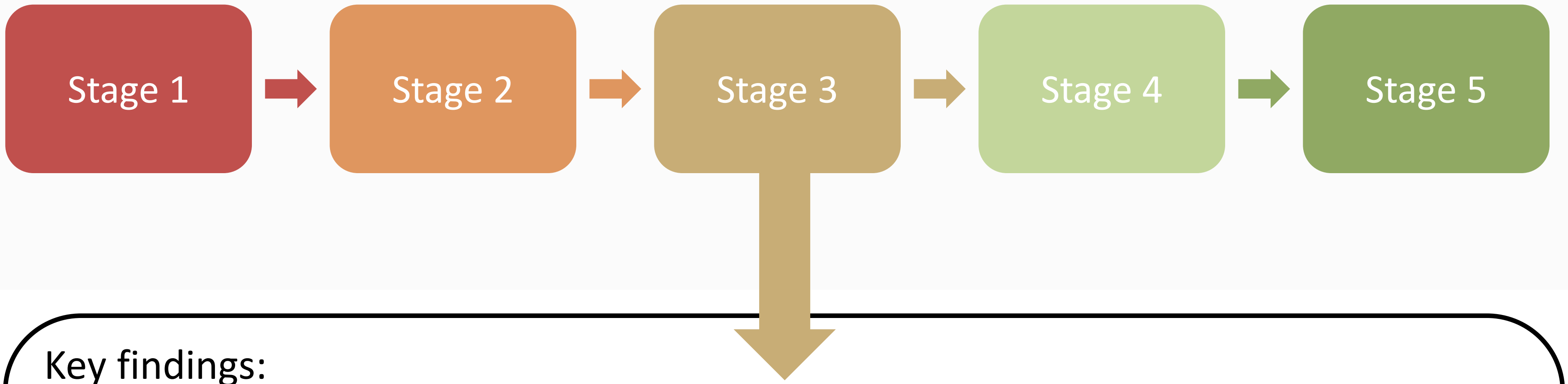

Key findings:

- Adherence was not routinely measured and some HCPs lacked the confidence and skills to manage adherence
- 8 important TDF domains: Knowledge, Skills, Beliefs about capability, Beliefs about consequences, Motivation, Social, Behavioural regulation, Nature of behaviour
- 26 BCTs identified that could potentially change healthcare professionals' ability to manage adherence

Stage 4: Identifying the existing evidence base

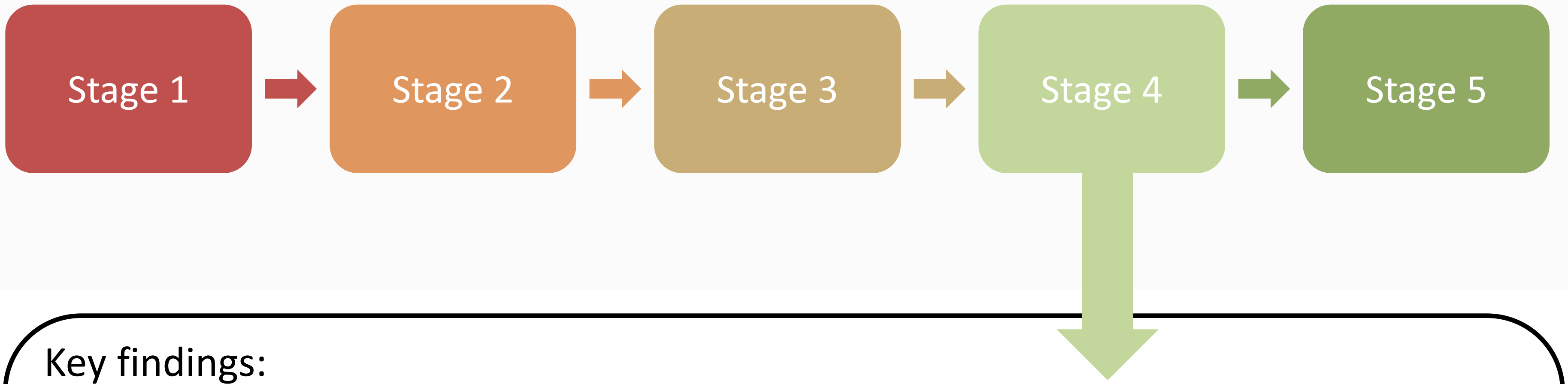

Key findings:

- 51 included studies
- 16 studies deemed interventions effective:
  - psychological frameworks used
  - multiple components
  - practical strategies to change adherence behaviour
  - individualised
  - no clear evidence on best way to deliver intervention

Stage 5: Triangulation of Stages 1 to 4

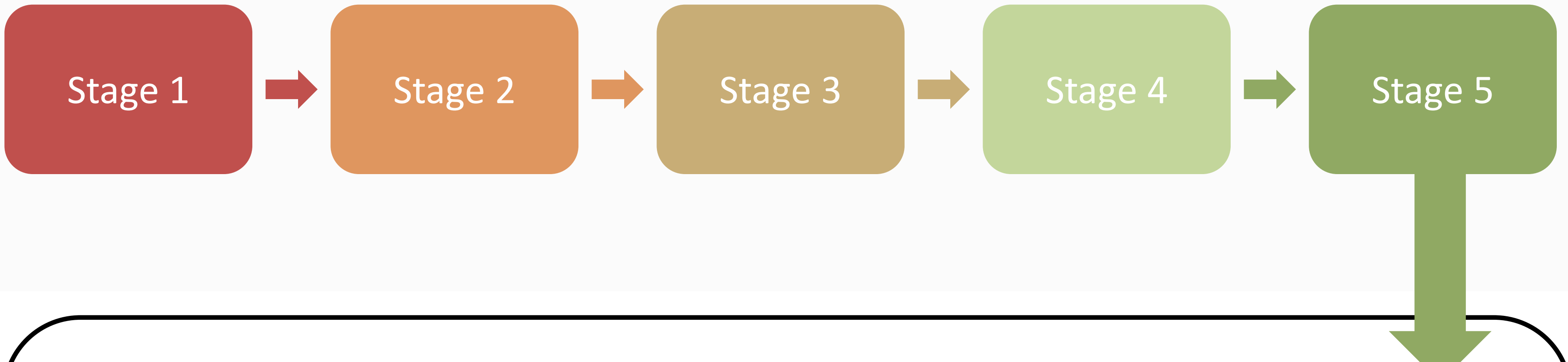

Key findings:

- Draft bronchiectasis intervention developed which includes:
  - 12 BCTs to change patients' adherence behaviour
  - bronchiectasis-specific content generated for each BCT
  - BCTs can be tailored to specific treatments
- Input needed from experts to determine how to deliver this intervention

Content of the intervention

Monitoring

Self-monitoring

Feedback

Information about behaviour

Social support

- ‘Toolkit’
- Tailored to individual patients
- Tailored to specific treatment

Persuasive communication

Role play

Cognitive restructuring

Graded task

Action planning

Goal specified

Problem-solving

What you might do with a patient

"Sometimes, I just can't be bothered (taking inhaled antibiotics) *and I know I should…I do feel better, overall, when I take it and I know I need to take it. But I just, sometimes, just can't be bothered at night time going through the whole rigmarole." (F11NA)*

BCTs used:

- Self-monitoring
- Problem-solving
- Goal setting
- Action planning

What you might do with a patient

"If I don't feel I've got it (mucus)*, I don't use the Acapella*® (a handheld device used to assist with the clearance of excess mucus from the airways)*." (M5NA)*

BCTs used:

- Persuasive communication
- Information about behaviour/outcome
- Self-monitoring

Summary

- Evidence-based approach to intervention development
- 5 stages of development involving key stakeholders and existing literature
- Intervention content:
  - 'Toolkit' of 12 BCTs
  - Tailored to individual patients
  - Tailored to specific treatments

'All medicine, all illness is personal to that individual'

- We know **what** will be included, now we need to know **how** to do it

What do you think about our approach?

Tea break

Queen's University Belfast and Ulster University logos

Development of a bronchiectasis-specific adherence intervention:

Session 2: Small group discussion

Dr Amanda McCullough

May 1st 2014

Outline of small group task

- 3 groups and 1.5 hours
- Format and delivery of intervention (Group 1): (names of participants)
- Training healthcare professionals (Group 2): (names of participants)
- Commissioning interventions (Group 3): (names of participants)

Lunch

Queen's University Belfast and Ulster University logos

Development of a bronchiectasis-specific adherence intervention:

Session 3: Feedback on small group discussion

May 1st 2014

Feedback on small group task

- Format and delivery of intervention (Group 1): (names of participants)
- Training healthcare professionals (Group 2): (names of participants)
- Commissioning interventions (Group 3): (names of participants)

Queen's University Belfast and Ulster University logos

Development of a bronchiectasis-specific adherence intervention:

Session 4: Close

Dr Amanda McCullough

May 1st 2014

Future plans

- Summarise feedback and send to you for comment
- Know the content of intervention and how to deliver it
- Develop intervention materials
- Feasibility/pilot testing
- Full randomised controlled trial

Questions?

Thank you

Funders

Funder logos

Acknowledgements

Dr Michael Tunney, Prof. Alexandra Quittner, Dr Ian Bradbury and all of the staff and patients who participated in this research

FACILITATOR GUIDE

Meeting agenda

9.30-10.00am	Refreshments and consent
10.00-10.30am	Welcome and introduction
10.30-11.10am	Background to adherence intervention development and outline of patient adherence intervention
11.05-11.15am	Task 1
11.15-11.30am	**Coffee**
11.30-1.00pm	Task 2: Small group session: *Defining how the patient adherence intervention could be delivered and how healthcare professionals could be trained to deliver it*
1.00pm-1.45pm	**Lunch**
1.45pm-3.30pm	Task 3: Feedback and discussion from the small group session
3.30-4.00pm	Close

Task 1: Consensus on methodological approach used (5-10 minutes)

Present the methodology used to develop this intervention during the 'background' presentation – have a short general discussion and agree credibility of work so far.

Task 2: Defining how the patient intervention could be delivered and how healthcare professionals could be trained to deliver it (1.5 hours)

Split panel into 3 groups (AMcC):

Group 1: Format and delivery of the intervention (Facilitators: AMcC and JB)

Panel members: (names)

Resources needed:

- List of patient BCTs and examples
- Summary of relevant TDF domain findings (Patient/HCP behavioural regulation)
- List of questions

Facilitator role: Ask panel members to review the BCTs, examples and TDF data provided and discuss the following questions. They should brainstorm ideas, nominate a note-taker who will record their views on the flip chart provided and agree a person to feed back to the group. Facilitator should provide these instructions, leave the group for 15 to 20 minutes to allow discussion, then go back to the group to review progress and facilitate discussion. Facilitator should ensure groups are recording answers to questions and have covered all questions during the allocated time.

1. Which patients should the intervention be delivered to?
2. Who should deliver the intervention? *(Facilitator prompts: type of healthcare professional, other)*
3. How often should the intervention be delivered?
4. For how long should the intervention be delivered?
5. What format should the intervention take? *(Facilitator prompts: written, verbal, web-based, group, one-to-one)*
6. Where should the intervention be delivered? *(Facilitator prompts: home, in-patient, out-patient, primary/secondary care)*

Group 2: Training healthcare professionals (Facilitators: CH and CR)

Panel members: (names)

Resources needed:

- List of patient BCTs and examples
- Summary of relevant TDF domain findings (Patient/HCP behavioural regulation, HCP knowledge/skills/capabilities)
- List of questions

Facilitator role: Ask panel members to review the BCTs, examples and TDF data provided and discuss the following questions. They should brainstorm ideas, nominate a note-taker who will record their views on the flip chart provided and agree a person to feed back to the group for the two tasks below. Facilitator should provide these instructions, leave the group for 15 to 20 minutes to allow discussion then go back to the group to review progress and facilitate discussion. Facilitator should ensure groups are recording answers to questions and have covered all questions during the allocated time.

1. Which healthcare professionals should the training be delivered to? (e.g. different healthcare professionals required training)
2. Who should deliver the healthcare professional training? (e.g. healthcare professional within the team, someone outside the team)
3. How often should the training take place?
4. How long should the training be?
5. What format should the healthcare professional training take? (e.g. written, verbal, web-based, group, one-to-one)
6. Where should healthcare professional training be delivered? (e.g. remotely or out-patient, in-patient)

Group 3: Commissioning interventions (Facilitators: BO'N and SE):

Panel members: (names)

Resources needed:

- List of patient BCTs and examples
- Summary of relevant TDF domain findings (environment)
- List of questions

Facilitator role: Ask panel members to review the BCTs, examples and TDF data provided and discuss the following questions. They should brainstorm ideas, nominate a note-taker who will record their views on the flip chart provided and agree a person to feed back to the group for the two tasks below. Facilitator should provide these instructions, leave the group for 15 to 20 minutes to allow discussion then go back to the group to review progress and facilitate discussion. Facilitator should ensure groups are recording answers to questions and have covered all questions during the allocated time.

1. How do you commission services at the moment?
2. How do you commission training for staff at the moment?
3. Would improved adherence be enough to convince you that this intervention was worth implementing?
4. What would you need to convince you that this intervention was worth implementing?

Task 3 (1.75 hours)

Each group presents their feedback on their respective questions (30-45 minutes per group). Group facilitators lead feedback session whilst another facilitator takes notes.

Format and delivery of intervention: AMcC to facilitate feedback (15 mins) and discussion (15 mins); Cris to take notes.

Training healthcare professionals: Carmel and Cris to facilitate feedback (15 mins) and discussion (15 mins); Amanda to take notes.

Commissioning interventions: Brenda to facilitate feedback (15 mins) and discussion (20 mins); Amanda to take notes.

Group 1: Format and delivery of intervention

Panel members: (names)

Please read the information that you have been given about the intervention. Please read the questions below and discuss them with the group. Please nominate someone to record your answers on the flip chart provided and agree a person to feed back to the rest of the group.

1. Which patients should the intervention be delivered to?
2. Who should deliver the intervention?
3. How often should the intervention be delivered?
4. How long should the intervention be delivered for?
5. What format should the intervention take?
6. Where should the intervention be delivered?

Summary of patient and healthcare professional *'Behavioural Regulation'* domain

Definition: Includes the 'how' of changing clinical practice: what are the practical strategies that would facilitate or hinder uptake of a new practice.

Patients thought that the intervention could:

- Be led by a knowledgeable healthcare professional.
- Be reinforced by different healthcare professionals.
- Include reviews every 3 months that could be increased or decreased depending on what patients needed.
- Be either one-to-one with their healthcare professional or in a group.
- Include information that was written, verbal or shown on posters or diagrams.
- Include information which uses everyday language that patients could understand

Healthcare professionals thought that the patient intervention could:

- Be tailored to individual patients' needs at different times during their disease.
- Prioritise sicker patients or those who were struggling to do treatments.
- Include healthcare professionals working as a multidisciplinary team including GPs and hospital healthcare professionals.
- Include information that was written, verbal or shown on posters or diagrams.
- Include information which uses everyday language that patients could understand
- Be either one-to-one with healthcare professional or in a group. They thought groups may be better in a hospital setting.
- To overcome limited time to deliver the intervention, some healthcare professionals suggested having regular review and/or multidisciplinary clinics.

Regarding training, healthcare professionals reported that:

- Secondary care professionals could provide bronchiectasis education to GPs and practice nurses as part of ongoing GP training programmes.

- Consultation skills training could be delivered to the whole multidisciplinary team but that mentorship would be needed. One psychologist interviewed stated that this is something she would be qualified to deliver.

Group 2: Training healthcare professionals

Panel members: (names)

Please read the information that you have been given about the intervention. Please read the questions below and discuss them with the group. Please nominate someone to record your answers on the flip chart provided and agree a person to feed back to the rest of the group.

1. Which healthcare professionals should the training be delivered to?
2. Who should deliver the healthcare professional training?
3. How often should the training be?
4. How long should the training be?
5. What format should the healthcare professional training take?
6. Where should healthcare professional training be delivered?

Summary of patient and healthcare professional *'Behavioural Regulation'* domain

Definition: Includes the 'how' of changing clinical practice: what are the practical strategies that would facilitate or hinder uptake of a new practice.

Patients thought that the intervention could:

- Be led by a knowledgeable healthcare professional.
- Be reinforced by different healthcare professionals.
- Include reviews every 3 months that could be increased or decreased depending on what patients needed.
- Be either one-to-one with their healthcare professional or in a group.
- Include information that was written, verbal or shown on posters or diagrams.
- Include information which uses everyday language that patients could understand

Healthcare professionals thought that the patient intervention could:

- Be tailored to individual patients' needs at different times during their disease.
- Prioritise sicker patients or those who were struggling to do treatments.
- Include healthcare professionals working as a multidisciplinary team including GPs and hospital healthcare professionals.
- Include information that was written, verbal or shown on posters or diagrams.
- Include information which uses everyday language that patients could understand
- Be either one-to-one with healthcare professional or in a group. They thought groups may be better in a hospital setting.
- To overcome limited time to deliver the intervention, some healthcare professionals suggested having regular review and/or multidisciplinary clinics.

Regarding training, healthcare professionals reported that:

- Secondary care professionals could provide bronchiectasis education to GPs and practice nurses as part of ongoing GP training programmes.
- Consultation skills training could be delivered to the whole multidisciplinary team but that mentorship would be needed. One psychologist interviewed stated that this is something she would be qualified to deliver.

Summary of healthcare professional *'Knowledge'* domain

Definition: Knowledge of the field (i.e. whether there is adequate evidence) and individuals' knowledge of the evidence or of a guideline

Healthcare professionals reported that:

- They would like access to updated bronchiectasis training.
- There was a lack of knowledge about bronchiectasis and its management amongst primary care participants.
- Specialist secondary care professionals considered themselves to be knowledgeable about bronchiectasis and its management. They were aware of a lack of knowledge of primary care professionals about the management of bronchiectasis.
- They had a broad understanding of the potential barriers for patients to adherence to treatment and were aware of adherence issues such as underuse and overuse of medication. They gained most of their knowledge about adherence from past experience with patients.
- Knowing their patients was key to being able to manage adherence to treatment.

Summary of healthcare professional *'Skills'* domain

Definition: Covers the possibility that new skills would be required by the staff who are required to implement a new procedure

Healthcare professionals reported that:

- Communication/interpersonal skills were key to managing adherence, particularly the ability to build up rapport, honesty and trust with patients.
- Negotiation, problem solving and persuasive communication skills could be used to assist patients to adhere to treatment.
- Some participants felt that they lacked skills in questioning about adherence and were concerned about creating confrontation.
- Some participants also felt they lacked the skills to manage adherence difficulties and wanted training on techniques such as cognitive behavioural therapy and advanced communication skills.
- A minority had had formal postgraduate training in communication skills which they felt benefitted them in being able to challenge patients about adherence and manage adherence issues, whereas others learnt these skills through experience.
- Specialist healthcare professionals did not report any skill deficits in assessment or treatment of bronchiectasis and felt they were able to manage the condition appropriately.
- Specialist healthcare professionals noted that GPs may require more training in diagnostic/monitoring skills of people who may have bronchiectasis to be able to give those people appropriate diagnosis and referral to secondary care.

Summary of healthcare professional *'Beliefs about capabilities'* domain

Definition: How confident clinicians are that they could change their practice effectively

Healthcare professionals reported that:

- They had a general belief that 'there was only so much you can do' to control patients' adherence behaviour.
- In some cases, they lacked confidence in their ability to question patients and manage problems with adherence without losing patients' trust.
- Others felt confident in their ability to manage adherence and those who did, tended to have completed extended communication skills training.
- Several participants appeared pessimistic about their ability to change their own behaviours regarding measuring, challenging and managing adherence to treatment. This lack of confidence was mainly linked to time and workload constraints.
- Several practice nurses indicated a lack of perceived competence in the management of bronchiectasis.

Group 3: Commissioning interventions

Panel members: (names)

Please read the information that you have been given about the intervention. Please read the questions below and discuss them with the group. Please nominate someone to record your answers on the flip chart provided and agree a person to feed back to the rest of the group.

1. How do you commission services at the moment?
2. How do you commission training for staff at the moment?
3. Would improved adherence be enough to convince you that this intervention was worth implementing?
4. What would you need to convince you that this intervention was worth implementing?

Summary of healthcare professional *'Environmental context'* domain

Definition: Includes the physical (including financial) issues that may limit change, including staffing levels and time as well as equipment or space.

Healthcare professionals reported that:

- Bronchiectasis services in primary care are not incentivised by the Quality and Outcomes Framework (QOF) and in secondary care all participants reported running unfunded services.
- Both primary and secondary care participants described working in a 'cost-saving' environment.
- Lack of funding was perceived to lead to pressures on staff time and workload, which were key barriers to managing patient adherence.
- Lack of access to appropriate equipment including nebulisers, Acapella® and easier preparations of inhaled antibiotics were thought to be barriers to managing adherence.
- Lack of access to specialist staff, in particular, community-based respiratory physiotherapists and psychologists were viewed by some as being a barrier to being able to manage adherence.
- System barriers included a lack of integration between primary and secondary care services e.g. integration of GP and pharmacy systems with hospital systems and vice versa.
- In primary care, patients were often not coded as bronchiectasis on GP systems, making it difficult to highlight these patients for review.
- In secondary care, a lack of multidisciplinary clinics was felt to be a barrier to managing adherence.
- Participants in secondary care described getting training in bronchiectasis as a 'battle.'
- Primary care professionals recognised that training in bronchiectasis management was not a priority for GPs or practice nurses or in the undergraduate degrees for pharmacy and medicine.

Table 1. Content of bronchiectasis-specific adherence intervention

We have used our research to date to design the content of the intervention. The intervention contains 12 different behavioural change techniques (BCTs). We envisage that this intervention will take the form of a 'toolkit.' This means that you can choose a number of BCTs from the list based on your patients' needs; thereby, allowing you to tailor the intervention to individual patients and to specific treatments.

Please read the table below. The first column outlines the 12 BCTs that could be used, along with their definition. The second column describes what you, as a healthcare professional, could do with your patient if you used a specific BCT. The information in the second column is one example of what you could do for each BCT. These examples can be changed to suit your patients' needs.

Behavioural change technique	What you would do with your patient
**Monitoring:** observe or record behavior with the person's knowledge as part of a behavior change strategy	Healthcare professional collects data on patient's non-adherence to specific treatment and health outcomes e.g. pulmonary exacerbations, lung function during a specific time period.
**Self-monitoring:** record own specified behaviour	Dependent on target i.e. medication vs ACT. Ask patient to record daily in a diary and record how often/how long treatment/medication. Ask patient to record relevant symptoms at the end of each day over the monitoring period e.g. one week.
**Feedback:** of monitored (including self-monitored behaviour)	Inform the patient of their level of adherence to a specific treatment and their number of pulmonary exacerbations during a given time period e.g. past year.
**Information regarding behaviour, outcome:** provide information about antecedents or consequences of the behaviour, or connections between them, or behaviour change techniques.	Explain the link between non-adherence and hospitalisation/increase in antibiotics and other therapies and increase in symptoms.
**Persuasive communication:** credible source presents arguments in favour of the behaviour. Note, there must be evidence of presentation of arguments; general pro-behaviour communication does not count.	Consider expert patients/Lead Consultant endorsing the importance of medication/ACT

Behavioural change technique	What you would do with your patient
**Cognitive restructuring:** changing cognitions about causes and consequences of behaviour. Suggest the deliberate adoption of a perspective or new perspective on behaviour (e.g. its purpose) in order to change cognitions or emotions about performing the behaviour.	Target specific medication/ACT.<br>Suggest that patients would think of tasks to reduce non-adherence to a specific medication/ACT.
**Problem-solving:** analyse , or prompt the person to analyse, factors influencing the behavior and generate or select strategies that include overcoming barriers and/or increasing facilitators (includes 'Relapse Prevention' and 'Coping Planning')	Identify specific barriers to adherence and rehearse strategies to overcome these barriers. Identify what could be done if there is non-adherence for some reason.
**Goal/target specified: behaviour or outcome:** set or agree on a goal defined in terms of the behaviour to be achieved	Agree a daily adherence goal (e.g. adhere once per day for a particular treatment) with the patient and reach agreement about the goal.
**Action planning: p**rompt detailed planning of performance of the behavior (must include at least one of context, frequency, duration and intensity). Context may be environmental (physical or social) or internal (physical, emotional or cognitive) (includes 'Implementation Intentions')	Create an action plan for the patient which should include: begin with target and then plan how you will achieve in terms of: days of week, time, where you will take medication/ACT. Identify barriers to action plan and strategies to overcome barriers. Rate (0-10) patient confidence in achieving action plan.
**Graded task, starting with easy tasks: s**et easy tasks to perform, making them increasingly difficult until target behaviour performed.	Consider target behaviour and consider baseline adherence. Ask patient to increase adherence to 1 day per week: week 1; 2 days per week: week 2 and so on until successfully approaching adherence.
**Demonstration/modelling (Role play):** perform behaviour in simulated situation.	Ask the patient to show you how they take their medication/perform ACT. Demonstrate the correct technique. Observe patient demonstrating it back to you.
**Social support:** Advise on, arrange or provide social support *(e.g. from friends, relatives, buddies' or healthcare staff)* or non-contingent praise or reward for performance of the behaviour.	Arrange for a family member (e.g. husband or wife) to encourage patient to continue with the behaviour change programme.
