# Supplementary material for: Defining the content and delivery of an intervention to Change AdhereNce to treatment in BonchiEctasis (CAN-BE): a qualitative approach incorporating the Theoretical Domains Framework, behavioural change techniques and stakeholder expert panels
Source: BMC Health Serv Res. 2015 Aug 22;15:342. doi: 10.1186/s12913-015-1004-z (PMC4546345; doi:10.1186/s12913-015-1004-z)
Supplement: Additional file 7: — Summary of patient expert panel findings. (PDF 1049 kb) [file 12913_2015_1004_MOESM7_ESM.pdf]

Queen's University Belfast and Ulster University logos

Research project: Development of a bronchiectasis-specific adherence intervention

Meeting title: Bronchiectasis meeting

Date: 10am on Monday 28th April 2014,

Location: Room N09/10, School of Pharmacy, Queen's University Belfast

SUMMARY OF DISCUSSION

Task 1: Does this plan look like something that would be helpful?

Most people were able to understand why it might be difficult to do treatments for bronchiectasis. They gave examples of times when they had difficulties taking treatments and the things they did to overcome these.

Everyone felt that this plan would be helpful for them. They felt that it could be easily used by lots of different people with bronchiectasis. They thought it could be easily tailored for different treatments e.g. airway clearance and oxygen.

Task 2: How can we use this plan in everyday life?

1. Which patients should get this intervention?

The group thought that everyone with bronchiectasis should get this plan. They thought that it could be tailored to how well or unwell they were. They did not think that it would be good to use this plan when they were very unwell e.g. during a severe chest infection.

2. Which healthcare professionals should deliver this intervention?

People thought that all healthcare professionals who care for those with bronchiectasis should be involved in some way. They thought that healthcare professionals at specialist bronchiectasis clinics, such as physiotherapists, specialist nurses and consultants might be the best people to deliver this plan. Some also thought that pharmacists might be able to deliver the plan. Most patients said they had a usual community pharmacist but did not regularly get information about medicines from them. They thought that GPs wouldn't have

time to do this plan with them but thought they should be involved in some way e.g. by being sent information about what was discussed with them as part of the intervention.

3. How often should you get this intervention?

People thought that this depended on how often they were seen at clinic. They thought that they should get the intervention a minimum of two or three times per year. They also thought that they might need a ‘top-up’ in between visits depending on whether they thought they needed more help. They thought that access to a ‘hotline’ might be one way you could do this.

4. How long should you get this intervention for?

People did not think that a one-off intervention would be useful. They thought that the intervention should be life-long.

5. What format should this intervention take?

People thought that a group with others with bronchiectasis might be the best way to do this. They thought that meeting other people with bronchiectasis in a group could be very encouraging and positive as they thought it would be helpful to share their experiences and learn from others. They thought that group size was important and that a small group of approximately six patients might be useful. They felt that they should be told about group size when they were invited to take part. Some people also said that there could be times where a one-to-one with a healthcare professional could be useful. They thought that they should be seen by the same healthcare professional during each visit.

People thought that personal stories from others with bronchiectasis had more impact on them. They thought these could be shared with them using a printed newsletter, an email, a YouTube video or by speaking to others as part of a group session.

6. Where should this intervention take place?

People said that group sessions should be located somewhere that they could easily get to e.g. hospital, community centre or health centre. They did not think meetings should be held on the same day as clinic visits as they are often unwell on those days. They thought that a few weeks after a clinic visit might be better.

7. How would you know if the intervention was working?

People said that they would know if the intervention was working if they had fewer chest infections or fewer admissions to hospital. They also said that they had individual ways of knowing if they were feeling better e.g. feeling in a better mood, being able to walk further, coughing less at night, snoring less, generally feeling better, family telling them they seemed in a better mood or take less medication e.g. Ventolin®.

Queen's University Belfast and Ulster University logos

Research project: Development of a bronchiectasis-specific adherence intervention

Meeting title: Bronchiectasis meeting

Date: 2.15pm on Monday 28th April 2014

Location: Room N09/10, School of Pharmacy, Queen's University Belfast

SUMMARY OF DISCUSSION

Task 1: Does this plan look like something that would be helpful?

Some people struggled to understand why it might be difficult to do treatments for bronchiectasis. Others felt that they had some difficulties taking treatments and gave examples of these and some of the things they did to overcome these e.g. self-management and pulmonary rehabilitation.

Everyone felt that this plan would be helpful for them. They felt that it could be easily used by lots of different people with bronchiectasis. They thought it could be easily tailored for different treatments e.g. airway clearance and inhaled antibiotics.

Task 2: How can we use this plan in everyday life?

1. Which patients should get this intervention?

The group thought that everyone with bronchiectasis should be offered this intervention and given the choice to decide whether to use it. Some people thought it would be less useful for those who did not have any difficulties taking treatments. They thought that access to this plan when they unwell would be useful. They did not think those who were terminally ill should get this plan.

2. Which healthcare professionals should deliver this intervention?

Everyone thought that specialist healthcare professionals, such as specialist nurses might be the best people to deliver this plan. However, they said that any healthcare professional (e.g. pharmacist or practice nurse) could deliver it as long as they had confidence in them

and had a good relationship with them. They did not think that GPs would have enough time to do this. They said that they were often treated by locum GPs who did not know their medical history and would not be able to deliver this intervention.

3. How often should you get this intervention?

Everyone thought that they should get a full review using the intervention once a year. They thought that some patients, such as older people, might need the intervention more frequently e.g. every 6 months. They thought that it would be useful to have reinforcement between annual visits but did not state how this could be done.

4. How long should you get this intervention for?

People did not think that a one-off intervention would be useful. They thought that the intervention should be life-long.

5. What format should this intervention take?

People thought that a group with others with bronchiectasis might be the best way to do this. They thought that meeting other people with bronchiectasis in a group could be very encouraging and positive as they thought it would be helpful to share their experiences and learn from others. They thought that the group should have between 15-20 people with bronchiectasis. They thought that the group could use a combination of written information, talks and personal stories from others with bronchiectasis. They said that a one-to-one visit with a nurse could be used before the group to discuss attendance at the group. They thought that family members could come to this one-to-one visit and also attend the group. They thought that they should be able to choose whether to bring their family members or not. They thought that written or verbal information could be given to family members to encourage patients to stick with changes in behaviour.

6. Where should this intervention take place?

Everyone said that group sessions should be located somewhere that they could easily get to e.g. hospital, community centre or health centre. They thought that a large room would be needed to accommodate people.

7. How would you know if the intervention was working?

People said that they would know if the plan was working if they had fewer chest infections or fewer admissions to hospital. They also said that they had individual ways of knowing if they were feeling better e.g. being in a better mood, being able to walk further, able to do

more around the house, generally feeling better, better peak flow or take less medications e.g. Ventolin®.
