# Supplementary material for: Defining the content and delivery of an intervention to Change AdhereNce to treatment in BonchiEctasis (CAN-BE): a qualitative approach incorporating the Theoretical Domains Framework, behavioural change techniques and stakeholder expert panels
Source: BMC Health Serv Res. 2015 Aug 22;15:342. doi: 10.1186/s12913-015-1004-z (PMC4546345; doi:10.1186/s12913-015-1004-z)
Supplement: Additional file 8: — Summary of HCP expert panel findings. (PDF 119 kb) [file 12913_2015_1004_MOESM8_ESM.pdf]

Research project: Development of a bronchiectasis-specific adherence intervention

Meeting title: Expert panel meeting

Date: 10am on Thursday 1st May

Location: Teaching Room, School of Geography, Queen's University Belfast

SUMMARY OF DISCUSSION

Task 1: What do you think about our approach?

The panel agreed that the approach that was used to develop the intervention was appropriate and had been carried out using a robust methodology. Panel members who worked with patients with bronchiectasis felt that the intervention was relevant for them and could be useful in clinical practice.

Task 2: How can we deliver this intervention?

Format and delivery of the intervention

1. *Which patients should the intervention be delivered to?*

The panel agreed that all patients with bronchiectasis should get the intervention. They thought that it should be offered as part of initial assessment. The panel described several different levels of intervention. Everyone should get a basic level of intervention and this could be intensified based on patient need e.g. those with worse adherence, more pulmonary exacerbations, mental health conditions or co-morbidities.

2. *Who should deliver the intervention?*

There was general agreement that there needed to be a lead healthcare professional to deliver the intervention to reduce repetition for patients. It was suggested that nurses or physiotherapists involved in bronchiectasis care could be best placed to do this. However, it was agreed that this may vary depending on the availability of these healthcare professionals. There were conflicting views on

the role of doctors in delivering this intervention. Panel members agreed that general practitioners (GPs) would not be able to deliver the intervention within existing service delivery models for bronchiectasis. However, they agreed that GPs should be informed of the delivery of the intervention to the patient. Panel members stated that if the intervention was delivered by a researcher that they should be integrated into the existing multidisciplinary team (MDT). Time constraints for all healthcare professionals were acknowledged.

3. *How often should the intervention be delivered?*

Panel members thought that the ‘basic’ intervention should be integrated into every interaction in secondary care. They agreed that the frequency of the intervention may need to be tailored to individual patient needs. Some suggested that a review at least annually was required whilst others thought that this left too long between visits.

4. *How long should the intervention be delivered for?*

Panel members thought that the intervention should become integrated into routine clinical care and be delivered on a continuous basis.

5. *What format should the intervention take?*

Panel members suggested that the intervention could be delivered using both one-to-one and group-based approaches. They thought that initial assessment/set-up of the intervention should be completed on a one-to-one basis with follow-up visits delivered in group format, with the ability to re-refer for one-to-one management. It was suggested that groups would contain only bronchiectasis patients, could be patient-led or have a clinician facilitator. Infection control was identified as a potential barrier to the use of this approach. Use of existing telehealth platforms was discussed. There was no agreement on the best way to use telehealth for this intervention.

6. *Where should the intervention be delivered?*

It was agreed that this intervention should take place predominantly within a secondary care hospital setting but that hospital staff could link with community respiratory teams who could undertake home visits. It was suggested that a separate ‘adherence clinic’ could be held; however, it was acknowledged that those who might be struggling with adherence may not attend additional visits. The panel thought that group-based interventions could be delivered in patients’ local areas e.g. in community centres or local hospitals.

Training of healthcare professionals

1. *Which healthcare professionals should the training be delivered to?*

The panel agreed that one lead healthcare professional in each site should be trained on the full intervention including behavioural change techniques (BCTs). It was agreed that all MDT members should receive a broader, less in-depth training on the intervention. The panel acknowledged that time constraints would be a barrier to this.

2. *Who should deliver the healthcare professional training?*

The panel agreed that a psychologist or another professional from outside the MDT who had expert knowledge in the delivery of BCTs should deliver the training.

3. *How often should the training be? How long should the training be?*

The panel agreed that there should be an initial half day session with the whole MDT. It was suggested that the lead healthcare professional would require additional incremental learning sessions. These could take the form of up to four, two-hour sessions. Up to three BCTs could be introduced in each session. Sessions could be two to three weeks apart, giving time to introduce new BCTs in between. During this practice period, mentoring or support would be needed for healthcare professionals to overcome any problems identified. This could take the form of email support or a telephone 'hotline.'

4. *What format should the healthcare professional training take?*

The panel agreed that problem-based learning in a group setting could be used for healthcare professionals. There were conflicting views on the use of online learning modules. Role plays and case studies were suggested ways of delivering this training.

5. *Where should healthcare professional training be delivered?*

The training should take place wherever is convenient for healthcare professionals.

Additional comments on the content of healthcare professional training

The group noted that the content of the healthcare professional had not been defined. They suggested using the same 12 BCTs when training healthcare professionals as those which you would use with patients. It was noted that generic training in BCTs exists but that the training package for this intervention would need to be tailored for this specific intervention covering topics such as how

the intervention was developed as well as the specific BCTs to be delivered to patients. It was also noted that there is medication adherence training delivered within Queen's University Belfast which uses motivational interviewing and cognitive behavioural therapy techniques.

Commissioning interventions

1. *How do you commission services at the moment?*

Panel members agreed that there were some existing systems in primary and secondary care that could be used for this intervention. It was noted that different commissioning streams exist in Northern Ireland and England and Wales. Services are funded in Northern Ireland through the use of Service Specifications and Frameworks. To able to obtain funding, the panel suggested that it would be important to get the intervention into the Respiratory Service Framework and built into the standards for bronchiectasis. Services can also be funded through a local enhanced services payment. In England and Wales, services are funded using the Tariff system. It was thought that the key issue would be getting the intervention into the Tariff system. In primary care in all localities, services are funded via the Quality and Outcomes Framework (QOF). It was recognised that bronchiectasis is not currently funded by QOF and therefore, this was unlikely to be a useful funding stream to pursue. Transforming Your Care (TYC) is a Northern Ireland-based initiative that provides non-recurrent funding for community-based services.

2. *How do you commission training for staff at the moment?*

Training for secondary care nursing staff in Northern Ireland is through the Department of Health, Social Services and Public Safety. Funding for training can also be tied into the Service Specifications/Frameworks. TYC provides non-recurrent funding for training of healthcare professionals in the community.

3. *Would improved adherence be enough to convince you that this intervention was worth implementing?*

The panel agreed that improvements in adherence would not be sufficient to convince commissioners that this intervention was worth implementing. It was acknowledged that better adherence could actually drive up medication costs and therefore, reductions in costs would have to evident elsewhere e.g. through reduced hospital admissions.

4. *What would you need to convince you that this intervention was worth implementing?*

The panel agreed that outcomes such as reductions in hospital admissions, healthcare usage, bed days, lost work days and improvements in quality of life drive commissioning. They also recognised that even with appropriate measurement of these outcomes, there are often delays in commissioning services.

Summary of participant feedback on expert panel summary document

Group 1

Q2 - Who should deliver intervention?

Enhanced adherence support might be best avoided from medical staff as there may be a risk of reporting adherence and barriers to explaining why adherence is not occurring

Q3 – How often should intervention take place?

I agree with the text here and this perhaps implicitly refers to the eventual intervention that will be rolled-out in the NHS. I would only add that the question was asked whether, for the purpose of the planned feasibility study and a possible subsequent pilot trial, you are trying to establish efficacy or effectiveness. It is often recommended that efficacy is established first and this might lead you to give the 'maximum' or most intensive intervention to all patients to give yourself the best chance of demonstrating proof-of-principle under ideal conditions. Effectiveness trials are often conducted once there is reasonable evidence of efficacy and this might be a trial of a more tailored version of the intervention where less severe patients received a less intensive intervention. This is a arguably a more feasible version of the interventions for healthcare systems t implement but it likely to result in smaller effect sizes. These are decisions for the researh team so you not feel you wish to include this in the document.

Additional comments – Measurement of intervention

Performance metrics of effective intervention probably needs a separate heading- its alluded to in the later section but I think we discussed it earlier too - we did not agree on how to measure efficacy of intervention!!!!!!!!!!!

Group 2

Under additional comments section

I agree with the text. I only note, as you already know, that MI and CBT are not BCTs but broad packages of (often not well specified) BCTs. In the context of a BCT approach to intervention development inclusion of any MI or CBT training element would require a further process of BCT specification otherwise you will introduce a confound into the evaluation since you will have lost a grip on the precise specification of your intervention.

Group 3

Q4

I agree with the text. I would note that for the feasibility study or a subsequent pilot RCT the primary outcome for what is essentially a complex behavioural intervention (i.e. shomehtig that is desinged to change behaviour) should be behavioural outcomes (i.e. nurses behaviour when delivering the training; patients' self-management behaviour) and not clinical (biometric) or service outcomes. These other outcomes become more important when you are conducting a later definiitive pragmatic-RCT.

General comments

I will attempt to give you a more in depth feedback later, however my first thoughts are that we also mentioned about a 'clinic' for more intense intervention which would perhaps be modelled on all the BCT's (whereas the annual, everyone model would perhaps be an group of these BCTs).

This clinic would then be for the more difficult bunch that you described. Also this clinic would be where the intervention was completed over a number of sessions alongside the team member where the adherence problems are more linked to.
